# A genomic catalog of Earth’s microbiomes

**DOI:** 10.1038/s41587-020-0718-6

**Published:** 2020-11-09

**Authors:** Stephen Nayfach, Simon Roux, Rekha Seshadri, Daniel Udwary, Neha Varghese, Frederik Schulz, Dongying Wu, David Paez-Espino, I-Min Chen, Marcel Huntemann, Krishna Palaniappan, Joshua Ladau, Supratim Mukherjee, T. B. K. Reddy, Torben Nielsen, Edward Kirton, José P. Faria, Janaka N. Edirisinghe, Christopher S. Henry, Sean P. Jungbluth, Dylan Chivian, Paramvir Dehal, Elisha M. Wood-Charlson, Adam P. Arkin, Susannah G. Tringe, Axel Visel, IMG/M Data Consortium, Helena Abreu, Silvia G. Acinas, Eric Allen, Michelle A. Allen, Lauren V. Alteio, Gary Andersen, Alexandre M. Anesio, Graeme Attwood, Viridiana Avila-Magaña, Yacine Badis, Jake Bailey, Brett Baker, Petr Baldrian, Hazel A. Barton, David A. C. Beck, Eric D. Becraft, Harry R. Beller, J. Michael Beman, Rizlan Bernier-Latmani, Timothy D. Berry, Anthony Bertagnolli, Stefan Bertilsson, Jennifer M. Bhatnagar, Jordan T. Bird, Jeffrey L. Blanchard, Sara E. Blumer-Schuette, Brendan Bohannan, Mikayla A. Borton, Allyson Brady, Susan H. Brawley, Juliet Brodie, Steven Brown, Jennifer R. Brum, Andreas Brune, Donald A. Bryant, Alison Buchan, Daniel H. Buckley, Joy Buongiorno, Hinsby Cadillo-Quiroz, Sean M. Caffrey, Ashley N. Campbell, Barbara Campbell, Stephanie Carr, JoLynn Carroll, S. Craig Cary, Anna M. Cates, Rose Ann Cattolico, Ricardo Cavicchioli, Ludmila Chistoserdova, Maureen L. Coleman, Philippe Constant, Jonathan M. Conway, Walter P. Mac Cormack, Sean Crowe, Byron Crump, Cameron Currie, Rebecca Daly, Kristen M. DeAngelis, Vincent Denef, Stuart E. Denman, Adey Desta, Hebe Dionisi, Jeremy Dodsworth, Nina Dombrowski, Timothy Donohue, Mark Dopson, Timothy Driscoll, Peter Dunfield, Christopher L. Dupont, Katherine A. Dynarski, Virginia Edgcomb, Elizabeth A. Edwards, Mostafa S. Elshahed, Israel Figueroa, Beverly Flood, Nathaniel Fortney, Caroline S. Fortunato, Christopher Francis, Claire M. M. Gachon, Sarahi L. Garcia, Maria C. Gazitua, Terry Gentry, Lena Gerwick, Javad Gharechahi, Peter Girguis, John Gladden, Mary Gradoville, Stephen E. Grasby, Kelly Gravuer, Christen L. Grettenberger, Robert J. Gruninger, Jiarong Guo, Mussie Y. Habteselassie, Steven J. Hallam, Roland Hatzenpichler, Bela Hausmann, Terry C. Hazen, Brian Hedlund, Cynthia Henny, Lydie Herfort, Maria Hernandez, Olivia S. Hershey, Matthias Hess, Emily B. Hollister, Laura A. Hug, Dana Hunt, Janet Jansson, Jessica Jarett, Vitaly V. Kadnikov, Charlene Kelly, Robert Kelly, William Kelly, Cheryl A. Kerfeld, Jeff Kimbrel, Jonathan L. Klassen, Konstantinos T. Konstantinidis, Laura L. Lee, Wen-Jun Li, Andrew J. Loder, Alexander Loy, Mariana Lozada, Barbara MacGregor, Cara Magnabosco, Aline Maria da Silva, R. Michael McKay, Katherine McMahon, Chris S. McSweeney, Mónica Medina, Laura Meredith, Jessica Mizzi, Thomas Mock, Lily Momper, Mary Ann Moran, Connor Morgan-Lang, Duane Moser, Gerard Muyzer, David Myrold, Maisie Nash, Camilla L. Nesbø, Anthony P. Neumann, Rebecca B. Neumann, Daniel Noguera, Trent Northen, Jeanette Norton, Brent Nowinski, Klaus Nüsslein, Michelle A. O’Malley, Rafael S. Oliveira, Valeria Maia de Oliveira, Tullis Onstott, Jay Osvatic, Yang Ouyang, Maria Pachiadaki, Jacob Parnell, Laila P. Partida-Martinez, Kabir G. Peay, Dale Pelletier, Xuefeng Peng, Michael Pester, Jennifer Pett-Ridge, Sari Peura, Petra Pjevac, Alvaro M. Plominsky, Anja Poehlein, Phillip B. Pope, Nikolai Ravin, Molly C. Redmond, Rebecca Reiss, Virginia Rich, Christian Rinke, Jorge L. Mazza Rodrigues, William Rodriguez-Reillo, Karen Rossmassler, Joshua Sackett, Ghasem Hosseini Salekdeh, Scott Saleska, Matthew Scarborough, Daniel Schachtman, Christopher W. Schadt, Matthew Schrenk, Alexander Sczyrba, Aditi Sengupta, Joao C. Setubal, Ashley Shade, Christine Sharp, David H. Sherman, Olga V. Shubenkova, Isabel Natalia Sierra-Garcia, Rachel Simister, Holly Simon, Sara Sjöling, Joan Slonczewski, Rafael Soares Correa de Souza, John R. Spear, James C. Stegen, Ramunas Stepanauskas, Frank Stewart, Garret Suen, Matthew Sullivan, Dawn Sumner, Brandon K. Swan, Wesley Swingley, Jonathan Tarn, Gordon T. Taylor, Hanno Teeling, Memory Tekere, Andreas Teske, Torsten Thomas, Cameron Thrash, James Tiedje, Claire S. Ting, Benjamin Tully, Gene Tyson, Osvlado Ulloa, David L. Valentine, Marc W. Van Goethem, Jean VanderGheynst, Tobin J. Verbeke, John Vollmers, Aurèle Vuillemin, Nicholas B. Waldo, David A. Walsh, Bart C. Weimer, Thea Whitman, Paul van der Wielen, Michael Wilkins, Timothy J. Williams, Ben Woodcroft, Jamie Woolet, Kelly Wrighton, Jun Ye, Erica B. Young, Noha H. Youssef, Feiqiao Brian Yu, Tamara I. Zemskaya, Ryan Ziels, Tanja Woyke, Nigel J. Mouncey, Natalia N. Ivanova, Nikos C. Kyrpides, Emiley A. Eloe-Fadrosh

**Affiliations:** 1 DOE Joint Genome Institute, Berkeley, CA USA; 2 Argonne National Laboratory, Argonne, IL USA; 3 Lawrence Berkeley National Laboratory, Berkeley, CA USA; 4 Present Address: Lawrence Berkeley National Laboratory, Berkeley, CA USA; 5 Travessa Alexandre da Conceicao, ALGAplus, Ilhavo, Portugal; 6 Department of Marine Biology and Oceanography, Institute of Marine Sciences-CSIC, Barcelona, Spain; 7 Scripps Institution of Oceanography, University of California, San Diego, La Jolla, CA USA; 8 School of Biotechnology and Biomolecular Sciences, UNSW Sydney, Sydney, New South Wales Australia; 9 Centre for Microbiology and Environmental Systems Science, University of Vienna, Vienna, Austria; 10 Department of Environmental Science, Aarhus University, Roskilde, Denmark; 11 Rumen Microbiology, Animal Science, AgResearch, Grasslands Research Centre, Palmerston North, New Zealand; 12 Department of Biology, Pennsylvania State University, University Park, Pennsylvania, PA USA; 13 Scottish Association for Marine Science, Oban, UK; 14 Department of Earth and Environmental Sciences, University of Minnesota, Minneapolis, MN USA; 15 Department of Marine Science, University of Texas Austin, Austin, TX USA; 16 Institute of Microbiology of the Czech Academy of Sciences, Praha 4, Czech Republic; 17 Department of Biology, University of Akron, Akron, OH USA; 18 Department of Chemical Engineering & eScience Institute, University of Washington, Seattle, WA USA; 19 Department of Biology, University of North Alabama, Florence, AL USA; 20 Life and Environmental Sciences and Sierra Nevada Research Institute, University of California, Merced, Merced, CA USA; 21 Ecole Polytechnique Federale de Lausanne, Lausanne, Switzerland; 22 Department of Soil Science, University of Wisconsin-Madison, Madison, WI USA; 23 School of Biological Sciences, Georgia Institute of Technology, Atlanta, GA USA; 24 Department of Aquatic Sciences and Assessment, Swedish University of Agricultural Sciences, Uppsala, Sweden; 25 Department of Biology, Boston University, Boston, MA USA; 26 University of Arkansas for Medical Sciences, Little Rock, AR USA; 27 Biology Department, University of Massachusetts Amherst, Amherst, MA USA; 28 Department of Biological Sciences, Oakland University, Rochester, MI USA; 29 Institute of Ecology and Evolution, University of Oregon, Eugene, OR USA; 30 Department of Soil and Crop Sciences, Colorado State University, Fort Collins, CO USA; 31 School of Geography and Earth Sciences, McMaster University, Hamilton, Ontario Canada; 32 School of Marine Sciences, University of Maine, Orono, ME USA; 33 Department of Life Sciences, Natural History Museum, London, UK; 34 LanzaTech., Skokie, IL USA; 35 Department of Oceanography and Coastal Sciences, Louisiana State University, Baton Rouge, LA USA; 36 Max Planck Institute for Terrestrial Microbiology, Marburg, Germany; 37 Deptartment of Biochemistry and Molecular Biology, The Pennsylvania State University, University Park, PA USA; 38 Department of Microbiology, University of Tennessee, Knoxville, TN USA; 39 Cornell University, Ithaca, NY USA; 40 Division of Natural Sciences, Maryville College, Maryville, TN USA; 41 School of Life Sciences, Arizona State University, Tempe, AZ USA; 42 University of Toronto, Toronto, Ontario Canada; 43 Lawrence Livermore National Laboratory, Livermore, CA USA; 44 Department of Biological Sciences, Clemson University, Clemson, SC USA; 45 Biology Department, Hartwick College, Oneonta, NY USA; 46 Akvaplan-niva, Fram—High North Research Centre for Climate and the Environment, Tromsø, Norway; 47 School of Science, University of Waikato, Hamilton, New Zealand; 48 Department of Soil, Water and Climate, University of Minnesota, Minneapolis, MN USA; 49 Biology Department, University of Washington, Seattle, WA USA; 50 Department of Chemical Engineering, University of Washington, Seattle, WA USA; 51 Department of the Geophysical Sciences, University of Chicago, Chicago, IL USA; 52 INRS-Centre Armand-Frappier Santé Biotechnologie, Laval, Quebec Canada; 53 Department of Chemical and Biomolecular Engineering, North Carolina State University, Raleigh, NC USA; 54 Environmental Microbiology Department, Instituto Antartico Argentino and Universidad de Buenos Aires, Buenos Aires, Argentina; 55 University of British Columbia, Vancouver, British Columbia Canada; 56 College of Earth, Ocean, and Atmospheric Sciences, Oregon State University, Corvallis, OR USA; 57 Department of Bacteriology, University of Wisconsin-Madison, Madison, WI USA; 58 Department of Microbiology, University of Massachusetts Amherst, Amherst, MA USA; 59 Department of Ecology and Evolutionary Biology, University of Michigan, Ann Arbor, MI USA; 60 Commonwealth Scientific Industrial Research Organisation, Brisbane, Queensland Australia; 61 College of Natural and Computational Sciences, Addis Ababa University, Addis Ababa, Ethiopia; 62 Laboratorio de Microbiología Ambiental, Centro para el Estudio de los Sistemas Marinos (CESIMAR, CONICET), Puerto Madryn, Argentina; 63 California State University, San Bernadino, San Bernardino, CA USA; 64 Department of Marine Microbiology and Biogeochemistry, NIOZ, Royal Netherlands Institute for Sea Research and Utrecht University, AB Den Burg, the Netherlands; 65 Department of Bacteriology, Wisconsin Energy Institute, University of Wisconsin-Madison, Madison, WI USA; 66 Centre for Ecology and Evolution in Microbial Model Systems (EEMiS), Linnaeus University, Växjö, Sweden; 67 Department of Biology, West Virginia University, Morgantown, WV USA; 68 Department of Biological Sciences, University of Calgary, Calgary, Alberta Canada; 69 J. Craig Venter Institute, La Jolla, CA USA; 70 Department of Ecosystem and Conservation Sciences, University of Montana, Missoula, MT USA; 71 Department of Geology and Geophysics, Woods Hole Oceanographic Institution, Woods Hole, MA USA; 72 Departments of Chemical Engineering and Applied Chemistry and Cell and Systems BIology, University of Toronto, Toronto, Ontario Canada; 73 Department of Microbiology and Molecular Genetics, Oklahoma State University, Stillwater, OK USA; 74 Visolis, Hayward, CA USA; 75 Great Lakes Bioenergy Research Center, University of Wisconsin-Madison, Madison, WI USA; 76 Department of Biology, Widener University, Chester, PA USA; 77 Department of Earth System Science, Stanford University, Stanford, CA USA; 78 Department of Ecology, Environment and Plant Sciences, Science for Life Laboratory, Stockholm University, Stockholm, Sweden; 79 Departments of Microbiology, The Ohio State University, Columbus, OH USA; 80 Texas A&M University, College Station, TX USA; 81 Human Genetics Research Center, Baqiyatallah University of Medical Sciences, Tehran, Iran; 82 Organismic and Evolutionary Biology, Harvard University, Cambridge, MA USA; 83 Department of Biomass Science and Conversion Technology, Sandia National Laboratory, Livermore, CA USA; 84 Ocean Sciences Department, University of California, Santa Cruz, Santa Cruz, CA USA; 85 Natural Resources Canada, Geological Survey of Canada, Calgary, Alberta Canada; 86 Graduate Group in Ecology, University of California, Davis, Davis, CA USA; 87 Department of Earth and Planetary Sciences, University of California, Davis, Davis, CA USA; 88 Lethbridge Research and Development Centre, Agriculture and Agri-Food Canada, Lethbridge, Alberta Canada; 89 Center for Microbial Ecology, Michigan State University, East Lansing, MI USA; 90 Department of Crop and Soil Sciences, University of Georgia Griffin Campus, Griffin, GA USA; 91 Department of Microbiology and Immunology, University of British Columbia, Vancouver, British Columbia Canada; 92 Department of Chemistry and Biochemistry, Thermal Biology Institute, and Center for Biofilm Engineering, Montana State University, Bozeman, MT USA; 93 Centre for Microbiology and Environmental Systems Science, Department of Microbiology and Ecosystem Science, Division of Microbial Ecology, University of Vienna, Vienna, Austria; 94 Department of Civil and Environmental Engineering, University of Tennessee, Knoxville, TN USA; 95 School of Life Sciences, University of Nevada, Las Vegas, Las Vegas, NV USA; 96 Research Center for Limnology (LIPI), Indonesian Institute of Sciences, Division of Inland Waterways Dynamics, Cibinong-Bogor, Indonesia; 97 Center for Coastal Margin Observation & Prediction (CMOP), Oregon Health & Science University, Portland, OR USA; 98 Biotechnological Management of Resources Network, Institute of Ecology, Xalapa, Mexico; 99 Department of Animal Science, University of California Davis, Davis, CA USA; 100 Diversigen, Houston, TX USA; 101 Department of Biology, University of Waterloo, Waterloo, Ontario Canada; 102 Duke University Marine Laboratory, Beaufort, NC USA; 103 Biological Sciences Division, Earth and Biological Sciences Directorate, Pacific Northwest National Laboratory, Richland, WA USA; 104 AnimalBiome, Oakland, CA USA; 105 Institute of Bioengineering, Research Center of Biotechnology, Russian Academy of Sciences, Moscow, Russia; 106 Division of Forestry and Natural Resources, West Virginia University, Morgantown, WV USA; 107 Department of Chemical and Biomolecular Engineering, North Carolina State University, Raleigh, NC USA; 108 Donvis Ltd, Ashhurst, New Zealand; 109 Department of Molecular and Cell Biology, University of Connecticut, Mansfield, CT USA; 110 School of Civil and Environmental Engineering, Georgia Institute of Technology, Atlanta, GA USA; 111 School of Life Sciences, Sun Yat-sen University, Guangzhou, China; 112 Laboratorio de Microbiología Ambiental, Instituto de Biología de Organismos Marinos, Puerto Madryn, Argentina; 113 Geological Institute, Department of Earth Sciences, ETH Zürich, Zürich, Switzerland; 114 Departamento de Bioquímica, Instituto de Química, Universidade de São Paulo, São Paulo, Brazil; 115 Great Lakes Institute for Environmental Research, University of Windsor, Windsor, Ontario Canada; 116 Departments of Civil and Environmental Engineering, and Bacteriology, University of Wisconsin-Madison, Madison, WI USA; 117 Commonwealth and Scientific Industrial Research Organisation, Brisbane, Queensland Australia; 118 School of Natural Resources and the Environment, University of Arizona, Tucson, AZ USA; 119 School of Environmental Sciences, University of East Anglia, Norwich Research Park, Norwich, UK; 120 Exponent Consulting, Pasadena, CA USA; 121 Department of Marine Sciences, University of Georgia, Athens, GA USA; 122 Division of Hydrologic Sciences, Desert Research Institute, Reno, NV USA; 123 Microbial Systems Ecology, Department of Freshwater and Marine Ecology, Institute for Biodiversity and Ecosystem Dynamics, University of Amsterdam, Amsterdam, the Netherlands; 124 Oregon State University, Corvallis, OR USA; 125 School of Geographical Sciences, University of Bristol, Bristol, UK; 126 Departments of Chemical Engineering and Applied Chemistry and Cell and Systems BIology, University of Toronto, Toronto, Ontario Canada; 127 Department of Civil & Environmental Engineering, University of Washington, Seattle, WA USA; 128 Department of Plants, Soils and Climate, Utah State University, Logan, UT USA; 129 Department of Chemical Engineering, University of California, Santa Barbara, Santa Barbara, CA USA; 130 Department of Plant Biology, University of Campinas, Campinas, Brazil; 131 Microbial Resources Division, Research Center for Chemistry, Biology and Agriculture, University of Campinas, Campinas, Brazil; 132 Department of Geosciences, Princeton University, Princeton, NJ USA; 133 Institute for Environmental Genomics, University of Oklahoma, Norman, OK USA; 134 Department of Biology, Woods Hole Oceanographic Institution, Woods Hole, MA USA; 135 Novozymes, Durham, NC USA; 136 Centro de Investigacion y de Estudios Avanzados del IPN (CINVESTAV), Unidad Irapuato, Irapuato, Mexico; 137 Department of Biology, Stanford University, Stanford, CA USA; 138 Oak Ridge National Laboratory, Oak Ridge, TN USA; 139 Department of Microorganisms, Leibniz Institute DSMZ - German Collection of Microorganisms and Cell Cultures, Braunschweig, Germany; 140 Department of Forest Mycology and Plant Pathology, Science for Life Laboratory, Swedish University of Agricultural Sciences, Uppsala, Sweden; 141 Genomic and Applied Microbiology & Göttingen Genomics Laboratory, Institute of Microbiology and Genetics, Georg-August University of Göttingen, Göttingen, Germany; 142 Faculty of Biosciences, Norwegian University of Life Sciences, Ås, Norway; 143 Department of Biological Sciences, University of North Carolina Charlotte, Charlotte, NC USA; 144 Department of Biology, New Mexico Institute of Mining and Technology, Socorro, NM USA; 145 Microbiology Department, and Byrd Polar and Climate Research Center, The Ohio State University, Columbus, OH USA; 146 Australian Centre for Ecogenomics/School of Chemistry and Molecular Biosciences, University of Queensland, Brisbane, Queensland Australia; 147 Research Computing Division, Harvard Medical School, Boston, MA USA; 148 Division of Pulmonary Sciences and Critical Care Medicine, University of Colorado Denver, Anschutz Medical Campus, Aurora, CO USA; 149 Department of Biological Sciences, University of Cincinnati, Cincinnati, OH USA; 150 Department of Systems Biology, Agricultural Biotechnology Research Institute of Iran, Agricultural Research, Education, and Extension Organization, Karaj, Iran; 151 Department of Ecology and Evolutionary Biology, University of Arizona, Tucson, AZ USA; 152 Department of Civil and Environmental Engineering, University of Vermont, Burlington, VT USA; 153 University of Nebraska - Lincoln, Lincoln, NE USA; 154 Department of Microbiology and Molecular Genetics, Michigan State University, East Lansing, MI USA; 155 Faculty of Technology and Centrum for Biotechnology, Bielefeld University, Bielefeld, Germany; 156 California Lutheran University, Thousand Oaks, CA USA; 157 Departamento de Bioquímica, Instituto de Química, Universidade de São Paulo, São Paulo, Brazil; 158 Faculty of Science, University of Calgary, Calgary, Alberta Canada; 159 Life Sciences Institute, Deparment of Medicinal Chemistry, University of Michigan, Ann Arbor, MI USA; 160 Limnological Institute, Siberian Branch of the Russian Academy of Sciences, Irkutsk, Russia; 161 Department of Environmental Science, School of Natural Sciences, Technology and Environmental Studies, Södertörn University, Huddinge Municipality, Huddinge, Sweden; 162 Department of Biology, Kenyon College, Gambier, OH USA; 163 Genomics for Climate Change Research Center, University of Campinas, Campinas, Brazil; 164 Department of Civil and Environmental Engineering, Colorado School of Mines, Golden, CO USA; 165 Bigelow Laboratory for Ocean Sciences, East Boothbay, ME USA; 166 Departments of Microbiology and Civil, Environmental, and Geodetic Engineering, The Ohio State University, Columbus, OH USA; 167 National Biodefense Analysis and Countermeasures Center, Frederick, MD USA; 168 Department of Biological Sciences, Northern Illinois University, DeKalb, IL USA; 169 Department of Earth Science and Marine Science Institute, University of California, Santa Barbara, Santa Barbara, CA USA; 170 School of Marine & Atmospheric Sciences, Stony Brook University, Stony Brook, NY USA; 171 Department of Molecular Ecology, Max Planck Institute for Marine Microbiology, Bremen, Germany; 172 Department of Environmental Science, University of South Africa, Pretoria, South Africa; 173 Deptartment of Marine Sciences, University of North Carolina at Chapel Hill, Chapel Hill, NC USA; 174 Centre for Marine Science and Innovation & School of Biological, Earth and Environmental Sciences, University of New South Wales, Sydney, New South Wales Australia; 175 Department of Biological Sciences, University of Southern California, Los Angeles, Los Angeles, CA USA; 176 Department of Plant, Soil and Microbial Sciences, Michigan State University, East Lansing, MI USA; 177 Department of Biology, Williams College, Williamstown, MA USA; 178 University of Southern California, Los Angeles, Los Angeles, CA USA; 179 School of Chemistry and Molecular Biosciences, University of Queensland, Brisbane, Queensland Australia; 180 Departamento de Oceanografía & Instituto Milenio de Ocenografía, Universidad de Concepción, Bio Bio, Chile; 181 Department of Bioengineering, University of Massachusetts Dartmouth, Dartmouth, MA USA; 182 IBG-5, Karlsruhe Institute of Technology, Karlsruhe, Germany; 183 Ludwig-Maximilians-Universität München, Munich, Germany; 184 Department of Biology, Concordia University, Montreal, Quebec Canada; 185 School of Veterinary Medicine, Population Health and Reproduction, University of California, Davis, Davis, CA USA; 186 KWR Water Research Institute, Nieuwegein, the Netherlands; 187 Department of Biological Sciences, University of Wisconsin-Milwaukee, Milwaukee, WI USA; 188 Chan Zuckerberg Biohub, Stanford, CA USA; 189 Department of Civil Engineering, University of British Columbia, Vancouver, British Columbia Canada

**Keywords:** Computational biology and bioinformatics, Microbiology

## Abstract

The reconstruction of bacterial and archaeal genomes from shotgun metagenomes has enabled insights into the ecology and evolution of environmental and host-associated microbiomes. Here we applied this approach to >10,000 metagenomes collected from diverse habitats covering all of Earth’s continents and oceans, including metagenomes from human and animal hosts, engineered environments, and natural and agricultural soils, to capture extant microbial, metabolic and functional potential. This comprehensive catalog includes 52,515 metagenome-assembled genomes representing 12,556 novel candidate species-level operational taxonomic units spanning 135 phyla. The catalog expands the known phylogenetic diversity of bacteria and archaea by 44% and is broadly available for streamlined comparative analyses, interactive exploration, metabolic modeling and bulk download. We demonstrate the utility of this collection for understanding secondary-metabolite biosynthetic potential and for resolving thousands of new host linkages to uncultivated viruses. This resource underscores the value of genome-centric approaches for revealing genomic properties of uncultivated microorganisms that affect ecosystem processes.

## Abstract (web summary)

Cataloging microbial genomes from Earth’s environments expands the known phylogenetic diversity of bacteria and archaea.

## Main

A vast number of diverse microorganisms have thus far eluded cultivation and remain accessible only through cultivation-independent molecular approaches. Genome-resolved metagenomics is an approach that enables the reconstruction of composite genomes from microbial populations and was first applied to a low-complexity acid mine drainage community^1^. With advances in computational methods and sequencing technologies, this approach has now been applied at much larger scales and to numerous other environments, including the global ocean^2^, cow rumen^3^, human microbiome^4–6^, deep subsurface^7^ and aquifers^8^. These studies have led to substantial insights into evolutionary relationships and metabolic properties of uncultivated bacteria and archaea^8–10^.

Beyond expanding and populating the microbial tree of life^11,12^, a comprehensive genomic catalog of uncultivated bacteria and archaea would afford an opportunity for large-scale comparative genomics, mining for genes and functions of interest (for example, CRISPR–Cas9 variants^13^) and constructing genome-scale metabolic models to enable systems biology approaches^8,14,15^. Further, recent genome reconstructions of uncultivated bacteria and archaea have yielded unique insights into the evolutionary trajectories of eukaryotes and ancestral microbial traits^16–18^.

**Fig. 1:**
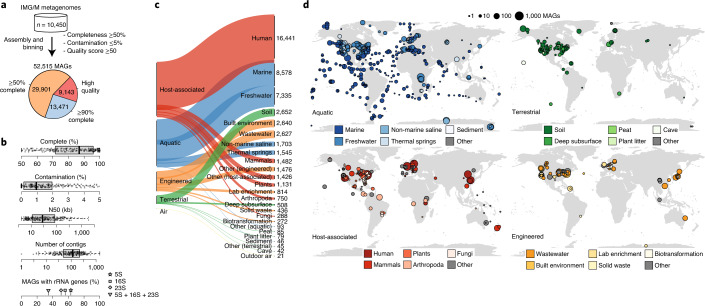
Environmental and geographic distribution of metagenome-assembled genomes. **a**, A total of 52,515 MAGs were recovered from geographically and environmentally diverse metagenomes in IMG/M. The majority (6,380 of 10,450; 61%) of metagenomes were reassembled for this work using the latest state-of-the-art assembly pipeline (Supplementary Table 1). These genomes form the GEM catalog. All MAGs were ≥50% complete, were ≤5% contaminated and had a quality score (completeness − 5 × contamination) of ≥50. **b**, Distribution of quality metrics across the MAGs. Approximately 200 randomly selected data points are overlaid on each boxplot, showing the minimum value, first quartile, median, third quartile and maximum value. See Supplementary Table 2 for quality statistics for all MAGs. **c**, Distribution of MAGs across biomes and sub-biomes, based on environmental metadata in the Genomes OnLine Database (GOLD; https://gold.jgi-psf.org). The number of MAGs associated with each sub-biome is indicated next to the plot. **d**, Geographic distribution of MAGs within each biome.

Here we applied large-scale genome-resolved metagenomics to recover 52,515 medium- and high-quality metagenome-assembled genomes (MAGs), which form the Genomes from Earth’s Microbiomes (GEM) catalog. The GEM catalog was constructed from 10,450 metagenomes sampled from diverse microbial habitats and geographic locations (Fig. 1). These genomes represent 12,556 novel candidate species-level operational taxonomic units (OTUs), representing a resource that captures a broad phylogenetic and functional diversity of uncultivated bacteria and archaea. To demonstrate the value of this resource, we used the GEM catalog to perform metagenomic read recruitment across Earth’s biomes, identify novel biosynthetic capacity, perform metabolic modeling and predict host–virus linkages.

## Results

### Over 52,000 metagenome-assembled genomes recovered from environmentally diverse metagenomes

We performed metagenomic assembly and binning on 10,450 globally distributed metagenomes from diverse habitats, including ocean and other aquatic environments (3,345), human and animal host-associated environments (3,536), as well as soils and other terrestrial environments (1,919), to recover 52,515 MAGs (Fig. 1a–c and Supplementary Tables 1 and 2). These metagenomes include thousands of unpublished datasets contributed by the Integrated Microbial Genomes and Microbiomes (IMG/M) Data Consortium, in addition to publicly available metagenomes (Methods and Supplementary Tables 1 and 2). This global catalog of MAGs contains representatives from all of Earth’s continents and oceans with particularly strong representation of samples from North America, Europe and the Pacific Ocean (Fig. 1d and Supplementary Fig. 1). The GEM catalog is available for bulk download along with environmental metadata (Data availability and Supplementary Table 1) and can be interactively explored via the IMG/M (https://img.jgi.doe.gov) or the Department of Energy (DOE) Systems Biology Knowledgebase (Kbase; https://kbase.us) web portals for streamlined comparative analyses and metabolic modeling.

MAGs from the GEM catalog all meet or exceed the medium-quality level of the MIMAG standard^19^ (mean completeness = 83%; mean contamination = 1.3%) and include 9,143 (17.4%) assigned as high quality based on the presence of a near-full complement of rRNAs, tRNAs and single-copy protein-coding genes (Fig. 1a,b and Supplementary Table 2). Genome sizes of high-quality GEMs ranged from 0.63 to 11.28 Mb, with most small-sized MAGs belonging to expected reduced genome lineages like the Nanoarchaeota or Mycoplasmatales, and similarly, large-sized MAGs belonging to Myxococcota and Planctomycetota. Genome size and GC content was lowest in host-associated microbiomes (median: 2.61 Mb; 46.9%) and highest in terrestrial microbiomes (median: 3.77 Mb; 57.1%), which is consistent with pangenome expansion in soil environments^20^. MAG sizes were consistent with isolate genomes of the same species, indicating no major loss of gene content in individual genomes (Supplementary Fig. 2). One exception was *Sinorhizobium medicae*, in which MAGs assembled from root nodules were nearly two times larger than isolate genomes (11–12 Mb compared to 6–7 Mb for isolate references; 99% average nucleotide identity (ANI) and 65% alignment fraction (AF) to *S. medicae* USDA1004). Although tetranucleotide frequency composition of binned scaffolds showed good consistency overall, numerous SNPs were detected, suggesting a composite arising from two strains of the same population. We additionally compared MAGs independently assembled by Parks et al.^10^ for a subset of GEM samples, which further reinforced the reproducibility of our composite genome bins (Supplementary Table 3 and Supplementary Note).

Taxonomically defined reference genomes are commonly used to infer the abundance of microorganisms from metagenomes but fail to recruit the majority of sequencing reads outside the human microbiome^21^. To explore whether the MAGs from the GEM catalog could address this issue, we aligned high-quality reads from 3,170 metagenomes with available read data to the 52,515 GEMs and to all isolate genomes from NCBI RefSeq. This revealed that an average of 30.5% (interquartile range (IQR) = 5.9–49.3%) and 14.6% (IQR = 0.9–15.8%) of metagenomic reads per sample were assigned to one or more GEMs or isolate genomes, respectively (Supplementary Fig. 3 and Supplementary Table 4). Across all samples, GEMs resulted in a median 3.6-fold increase in the number of mapped reads, which was particularly pronounced for certain environments like bioreactors or invertebrate hosts (Supplementary Fig. 3). Despite this improvement, nearly 70% of reads remained unmapped to any MAG or isolate genome. This was particularly noticeable for soil communities (for example, >95% of reads were unmapped to any genome in 55% of samples), which are highly complex and challenging to assemble^22,23^. Consistent with this result, metagenomes with the highest *k*-mer diversity^24^ tended to have the lowest mapping rates (Spearman’s *r* = −0.68; *P* value = 0). These communities likely contain closely related organisms, which pose a major problem for metagenomic assembly and binning^25^. Low mapping rates may also reflect the presence of viruses, plasmids and microbial eukaryotes, which were not recovered by the pipeline used in this study.

### The GEM catalog expands genomic diversity across the tree of life

**Fig. 2:**
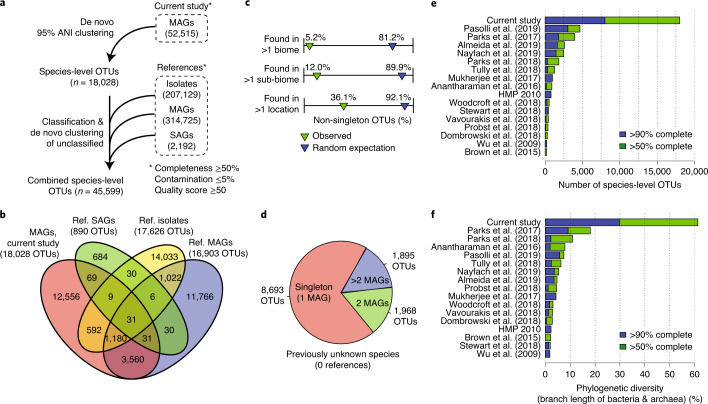
Species-level clustering of the GEM catalog with >500,000 reference genomes. **a**, MAGs from the current study were compared to 524,046 publicly available reference genomes found in IMG/M and NCBI. All reference genomes met the same minimum quality standards as applied to the GEM catalog. All MAGs and reference genomes were clustered into 45,599 species-level OTUs on the basis of 95% ANI and 30% AF. **b**, Overlap of OTUs between genome sets. MAGs from the current study revealed genomes for 12,556 species for the first time. **c**, The vast majority of OTUs with >1 genome from the GEM catalog were restricted to individual biomes and sub-biomes, although over a third were found in multiple geographic locations. **d**, A large proportion of the 12,556 newly identified species were represented by only a single genome. **e**,**f**, Comparison of the current dataset with the 16 largest previously published genome studies, selected on the basis of species-level diversity. Study identifiers were derived from either NCBI BioProject or GOLD. Studies by Wu et al. ^35^, HMP (2010)^36^ and Mukherjee at al. ^34^ contain additional genomes generated after publication. All MAGs from other studies were filtered using the same quality criteria as the GEM dataset (Fig. 1a and Methods). Genomes from the current study represent over three times more diversity compared to any previously published study.

To uncover new species-level diversity, we clustered GEMs on the basis of 95% whole-genome ANI revealing 18,028 species-level OTUs (Fig. 2a,b, Supplementary Fig. 4 and Supplementary Table 5). Although the species concept for prokaryotes is controversial^26^, this operational definition is commonly used and is considered to be a gold standard^27,28^. Based on taxonomic annotations from the Genome Taxonomy Database (GTDB)^29,30^, we found that the GEMs cover 137 known phyla, 305 known classes and 787 known orders. The vast majority of non-singleton OTUs contained GEMs from only a single environment or multiple closely related environments (for example, bioreactors and wastewater; Supplementary Fig. 5), suggesting that few species have a broad habitat range, whereas nearly 40% were found in multiple sampling locations (Fig. 2c). Accumulation curves of MAGs revealed no plateau for species-level OTUs (Supplementary Fig. 6), indicating that additional species remain to be discovered across biomes, which is also suggested from the low percentage of mapped reads.

Next, we compared the 18,028 OTUs against an extensive database of 524,046 reference genomes including >300,000 MAGs from previous studies, >200,000 genomes of organisms isolated in pure culture (including all of RefSeq) and >2,000 single-amplified genomes (SAGs; Fig. 2a). These included large MAG studies conducted in the human microbiome^4–6^, global ocean^2^, aquifer systems^7,8,31^, permafrost thaw gradient^14^, cow rumen^3^, hypersaline lake sediments^32^ and hydrothermal sediments^33^, as well as several large isolate genome sequencing studies such as the Genomic Encyclopedia of Bacteria and Archaea (GEBA) project^34,35^ and the Human Microbiome Project (HMP)^36^, although several studies were published during the course of the current study and were not included^37,38^. All reference genomes were subjected to the same quality criteria as we applied to the GEM dataset (≥50% completeness, ≤5% contamination and a quality score of ≥50).

Notably, 12,556 OTUs from the GEM catalog (representing 23,095 MAGs) were distinct from reference genomes at 95% ANI and thus represent new candidate species. At the same time, 70% of all reference genomes were recruited to the GEM catalog at >95% ANI, indicating it has good coverage of existing genomes. New OTUs were found in 326 studies, with an average of 40 for each study. The Microbial Dark Matter (MDM) Phase II study, an extension of the GEBA-MDM project^12^, contributed the most novelty with 790 new OTUs derived from 1,124 MAGs found in 80 metagenomes.

Supporting their novelty, the vast majority of the 12,556 new OTUs were distantly related to reference genomes or barely aligned at all (93.7% of OTUs with <90% ANI or <10% AF compared to references), and >99% were unannotated at the species level by the GTDB. However, MAGs from new OTUs tended to be slightly less complete (averages: 81.0% versus 84.6%), displayed slightly higher contamination (averages: 1.5% versus 1.1%) and were often found as singletons (Fig. 2d, Supplementary Table 6 and Supplementary Note). These observations are likely explained by a number of factors, including genome reduction for uncultivated lineages^6^, problems assembling the 16S rRNA locus^39^ and challenges recovering members of the rare biosphere^40^.

We clustered the unrecruited reference genomes into an additional 27,571 OTUs, resulting in a combined dataset of 45,599 species-level OTUs (Fig. 2a,b). This revealed that while the GEM catalog contained fewer genomes, it represented 3.8 times more diversity compared to any previously published study (Fig. 2e). For example, Parks et al. performed large-scale assembly and binning of all environmental metagenomes available in the NCBI Sequence Read Archive in an unprecedented effort to expand genomic representation of uncultivated lineages^10,30^. Based on the clustering and quality control performed in the current study, these 10,728 MAGs represent 5,200 OTUs, covering only 12% of OTUs from the GEM catalog (Supplementary Table 7).

**Fig. 3:**
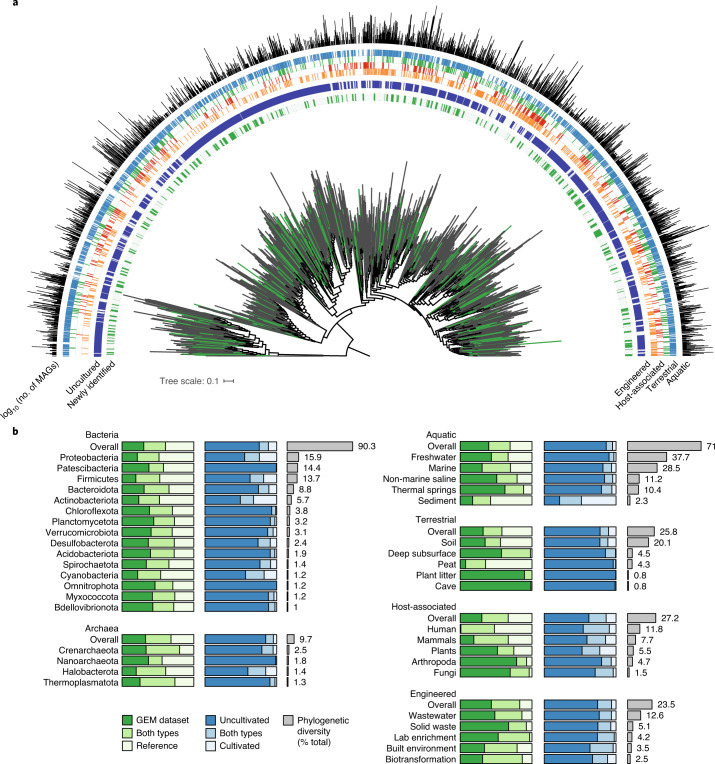
The GEM catalog fills gaps in the tree of life. **a**, A phylogenetic tree was built for 43,979 of the 45,599 OTUs based on a concatenated alignment of 30 universally distributed single-copy genes. The full alignment contained 4,689 amino acid positions, with each OTU containing data for at least 30% of positions. Species-level OTUs were further clustered based on phylogenetic distance into 1,928 approximately order-level clades. Green branches indicate new lineages represented only by the GEM catalog. The inner strip chart indicates whether an order is newly identified (green; represented only by GEMs) or was previously known (light gray; represented by a reference genome). The next strip chart indicates whether an order is uncultured (blue; represented only by MAGs/SAGs) or cultured (gray; represented by at least one isolate genome). The next four strip charts indicate the environmental distribution of the orders; the last plot indicates the number of MAGs from the GEM catalog recovered from each order. The GEM catalog’s composite genomes are broadly distributed across the tree of life, including many new order-level clades, though most new lineages are interspersed between existing ones. Vast regions of the tree are represented only by uncultivated genomes. **b**, Phylogenetic diversity was computed for subtrees represented by the GEM catalog/reference genomes (green scale) or cultivated/uncultivated genomes (blue scale). Gray bars indicate percentage of total phylogenetic diversity represented by each taxonomic group (left) or environment (right). The GEM catalog consistently expands phylogenetic diversity across different phyla within bacteria and archaea and for different environments. One exception is the human microbiome, where the GEM catalog contributes little new diversity. Combining the GEM catalog with other uncultivated genomes, it becomes apparent that uncultivated genomes dominate the diversity within most phyla and environments, particularly for groups like the Patescibacteria (Candidate Phyla Radiation) and Nanoarchaeota.

Next, we constructed a phylogeny of the 45,599 OTUs based on 30 concatenated marker genes (Fig. 3a, Supplementary Table 8 and Methods). Phylogenetic analysis of this tree supported that the GEM catalog is the most diverse dataset published to date (Fig. 2f). Overall, the GEM catalog resulted in a 44% gain in phylogenetic diversity across the entire tree of bacteria and archaea and currently represents 31% of all known diversity based on cumulative branch length. Gains in phylogenetic diversity were relatively consistent across taxonomic groups, but especially high for certain large clades that included Planctomycetota (79% gain), Verrucomicrobiota (68% gain) and Patescibacteria (also referred to as the ‘Candidate Phyla Radiation’) (60% gain) (Fig. 3b and Supplementary Table 9). The GEM catalog resulted in more variable gains across environments (Supplementary Table 10), though almost no new diversity was uncovered in human-associated samples (Fig. 3b) which were previously analyzed in recent MAG studies^4–6^. Notably, these analyses also revealed that 75% of the phylogenetic diversity of cataloged microbial diversity is exclusively represented by uncultured genomes (that is, MAGs or SAGs).

To determine whether the GEM catalog contained new lineages at higher taxonomic ranks, we used relative evolutionary divergence (RED)^30^ to cluster all 45,599 OTUs into monophyletic groups, including singletons, representing 16,062 genera, 5,165 families, 1,928 orders, 368 classes and 129 phyla (Supplementary Tables 11–13, Supplementary Fig. 7 and Methods). At the phylum level, we identified 16 clades exclusively represented by GEMs (11 clades in bacteria and 5 in archaea), which may indicate new phyla. However, these clades were supported by only 29 GEMs, which were largely assigned to known phyla by the tool GTDB-Tk (28/29). At lower taxonomic ranks, considerably more novel groups were identified, including 456 new orders, 1,525 new families and 5,463 new genera. We conclude that, in contrast to earlier metagenome binning studies that uncovered vast new lineages of life, the majority of deep-branching lineages are represented by current genome sequences.

### Encoded functional potential in the GEMs

To provide a systems-level snapshot of metabolic potential, we built genome-scale metabolic models for the nonredundant, high-quality GEMs with >40 representatives for each environment (*n* = 3,255) in KBase^41^ (Supplementary Figs. 8 and 9, Supplementary Table 14 and Supplementary Note). Beyond known metabolic pathways, we hypothesized that MAGs from the GEM catalog contained a reservoir of functional novelty. To address this question, we compiled a catalog of 5,794,145 protein clusters (PCs) representing 111,428,992 full-length genes, with 51.7% of PCs containing at least two sequences. The vast majority of PCs were not functionally annotated compared to the TIGRFAM or KEGG Orthology databases, and most lacked even a single Pfam domain (95.2%, 88.9% and 74.5% unannotated for TIGRFAM, KEGG and Pfam, respectively). Comparatively, for a catalog of 270 million genes from 76,000 reference bacterial and archaeal genomes available through IMG/M^42^, these percentages are approximately 70%, 50% and 20%, respectively. Nearly 70% of all PCs were not functionally annotated by any of the three databases, and 47% had no significant similarity to UniRef (https://www.uniprot.org), a large and regularly updated protein resource. While the largest PCs tended to be previously known, several large PCs lacked any annotation, including 356 clusters with at least 1,000 members and 28,869 clusters with at least 100 members.

While it is outside the scope of this study to systematically interpret the functional capacities of all GEMs, here we present a few illustrative vignettes. First, we found that GEMs recapitulated recent observations of an expanded purview of methanogenesis (Supplementary Fig. 10) due to membership of new archaeal phyla like the Halobacterota, Hadesarchaea (including Archaeoglobi and Syntrophoarchaeia) and lineages within the Crenarchaeota (for example, Thermoprotei, Korarchaeia and Bathyarchaeia)^43–46^. At a lower taxonomic rank, we identified GEMs for a novel species of the genus *Coxiella*, which includes the class B bioterrorism agent *Coxiella burnetii* associated with substantial health and economic burden^47^, providing an opportunity to gain new insights into the evolution of host–pathogen interactions within this genus. Several virulence factors were found in the GEMs, including the Dot/Icm type IV secretion system (Supplementary Fig. 7) used to deliver effector proteins into the cytoplasm of the host cell^48^; however, the characterized *C. burnetii* T4SS effectors were absent. Thus, GEMs offer potential for new discovery at the highest and lowest taxonomic ranks.

### Broad and diverse secondary-metabolite biosynthetic potential

**Fig. 4:**
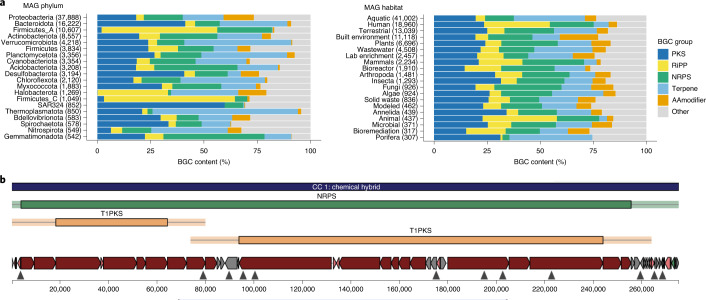
Biosynthetic gene clusters recovered from the GEMs dataset. **a**, Relative frequency of BGC types across dominant phyla (left) and habitats (right). BGC types are highly variable across phyla but relatively stable across habitats. AAmodifier, amino acid modifying system. **b**, The single largest BGC region, found in a soil-derived bacterium from the Acidobacteria phylum and UBA5704 genus. The BGC encodes 62 PKS or NRPS modules with three colinear module chains.

Most secondary metabolites have been isolated from cultivated bacteria affiliated to only a handful of bacterial groups, including*Streptomycetes*, *Pseudomonas*, *Bacillus* and *Streptococcus*^49^. More recently, mining of metagenomic data from soil has expanded representation to members of the phyla Acidobacteria, Verrucomicobia, Gemmatimonadetes and the candidate phylum Rokubacteria^50^. The GEM catalog affords a unique opportunity to explore the repertoire of secondary-metabolite biosynthetic gene clusters (BGCs) encoded within this taxonomically and biogeographically diverse genome collection. We identified 104,211 putative BGC regions from the 52,515 GEMs using AntiSMASH (v5.1)^51^ (Supplementary Table 15). For comparison, this represents an increase of BGCs in IMG/ABC (Atlas of BGCs)^52^ by 31% and is 54 times the size of the manually curated MIBiG dataset^49^. Approximately 66% of GEM BGCs intersected with one or more contig boundaries, indicating that a majority may be incomplete (Supplementary Fig. 12), which is consistent with previous observations based on fragmented recovery^50,53^. We assigned the class of secondary metabolites synthesized by each BGC across the GEM catalog (Fig. 4a). A total of 44,835 gene clusters or cluster fragments containing nonribosomal peptide synthetases (NRPSs) and/or polyketide synthases (PKSs) were identified from 104 phyla, 23,738 terpene clusters from 79 phyla and 12,360 ribosomally processed peptide (RiPP) clusters from 76 phyla. While fragmentation likely skewed cluster content counts in unpredictable ways, we observed trends that may be reflective of nature. For example, Firmicutes had unusually high numbers of RiPPs (more than half of their BGCs were RiPP clusters), while Thermoplasmatota and Verrucomicrobiota contained relatively high numbers of terpene clusters (68% and 50% of their BGCs, respectively). Analyses of environmental trends for BGCs were less clear, with no environmental source group showing a clear skew in relative BGC family content (Fig. 4a). If accurate, this implies that specific chemistry is not limited or amplified by environment, and that most classes of secondary metabolites can be found nearly anywhere.

To evaluate BGC novelty, we queried each BGC sequence against the NCBI nucleotide sequence collection. Using a threshold of 75% identity over 80% of the query length, we identified 87,187 (83%) as putatively novel BGCs that encoded new chemistry (Supplementary Table 16). Although many modular clusters are fragmented, we identified over 3,000 BGC regions >50 kb in length and more than 17,000 >30 kb. Together, the GEM catalog holds potential as a rich source of novel predicted BGCs and provides ample opportunity to explore biosynthetic potential outside known clades. As noted elsewhere^54^, *Myxococcus* showed promising biosynthetic potential, with 1,751 regions across 232 MAGs and a broad diversity of antiSMASH-defined BGC families. The single largest BGC region was found in a soil-derived bacterium putatively of the phylum Acidobacteria and genus UBA5704, encoding a remarkable number of 62 PKS or NRPS modules with three clear colinear module chains (Fig. 4b). Although several Acidobacteria are known to contain PKS and NRPS clusters, this MAG contains an additional 66 BGC regions, indicating a level of biosynthetic potential that may have been underestimated within this phylum.

### GEMs reveal thousands of new virus–host connections

In addition to the assembly of microbial genomes, recent studies have highlighted how metagenomes can be mined for novel viral genomes^55^. However, most uncultivated viruses cannot be associated with a microbial host, which is crucial for understanding their roles and impacts in nature. We reasoned that MAGs from the GEM catalog could be used to improve host prediction for viral genomes. To address this, we identified connections between the 52,515 GEMs and 760,453 viruses in IMG/VR^56^ using a combination of CRISPR-spacer matches (≤1 SNP) and genome sequence matches (>90% identity over >500 bp), which showed good agreement (Supplementary Note). IMG/VR viruses were connected to consistent host taxa (95% of linkages per virus to the same host family), and >96% of connected viruses and GEMs were derived from a similar environment based on the top level of the GOLD^57^ environmental ontology.

**Fig. 5:**
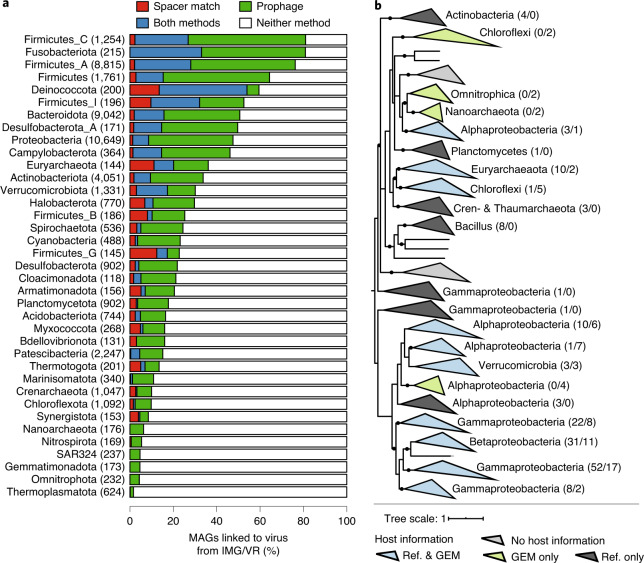
MAGs resolve host–virus connectivity. **a**, Bacterial and archaeal phyla from the GEM catalog were linked to viruses. The bar plot displays the percentage of MAGs linked to viruses from each phylum containing 100 or more MAGs. Phylum names were derived from the GTDB, and the numbers to the right represent MAGs from each phylum. Bar colors indicate the method of linking viruses to hosts; white indicates the percentage of MAGs not associated with any virus. **b**, Phylogeny of DJR viruses with associated host information. For each clade of three or more DJR sequences associated with the same host group, host information is indicated next to the clade along with the number of sequences linking this DJR clade to this host group, first from reference sequences, then from the GEM catalog. Reference sequences were obtained from Kauffman et al.^59^. Clades are colored according to the origin of the host information, and new host groups identified exclusively from the GEM catalog are highlighted in bold. All nodes with >50% support are displayed as multifurcation, and nodes with >80% support are highlighted with a black dot.

Using a combination of the two approaches, we predicted connections between 81,449 IMG/VR viruses and 23,082 GEMs (Fig. 5a and Supplementary Table 17), increasing the total number of IMG/VR viruses with a predicted host by >2.5-fold (from 36,976 to 92,872). However, these expanded virus–host connections still covered only 10.7% of the 760,453 viral genomes from IMG/VR and 44.0% of MAGs from the GEM catalog. This is exemplified for certain phyla like Thermoplasmatota, where a virus was linked to only 1.6% of the 624 assembled MAGs.

To address this limitation, we performed de novo prediction of integrated prophages in GEMs using VirSorter^58^ after carefully removing viral contamination (Methods). This approach provided an additional 10,410 viruses linked to 7,805 GEMs. These novel MAG-derived virus–host linkages included several groups of understudied clades, including the double jelly roll (DJR) lineage, which is a commonly overlooked group of non-tailed double-stranded DNA viruses^59,60^. Recent studies of DJR virus diversity have revealed that members of this group infect hosts across the three domains of life, yet they have also highlighted subgroups without a known host^59^. Here, we identified 73 DJR sequences in the GEM catalog, which provided host information for four additional DJR clades (Fig. 5b). In addition, two of these clades were linked through the GEMs to uncultivated bacterial and archaeal groups that had not yet been identified as putative DJR hosts (namely Omnitrophica and Nanoarchaeota). Beyond the DJR group, we identified putative hosts for two single-stranded DNA virus families, including four clades of *Microviridae* and 28 clades of *Inoviridae* (Supplementary Fig. 12 and Supplementary Table 18). Taken together, these different examples demonstrate how MAGs can resolve novel virus–host linkages.

## Discussion

This resource of 52,515 medium- and high-quality MAGs represents the largest effort to date to capture the breadth of bacterial and archaeal genomic diversity across Earth’s biomes. The GEM catalog considerably expands the known phylogenetic diversity of bacteria and archaea, increases recruitment of metagenomic sequencing reads, contains a wealth of biosynthetic potential and improves host assignments for uncultivated viruses. Despite an overall 44% increase in phylogenetic diversity of bacteria and archaea, we found little evidence of new deep-branching lineages representing new phyla, consistent with recent studies of microbial diversity^30,61^. Likewise, despite a 3.6-fold increase in recruitment of metagenomic reads, over two-thirds of metagenome reads still lack a mappable reference genome. Thus, continued efforts to capture the genomes of new species- and strain-level representatives will further improve metagenomic resolution.

Large-scale genomic inventories provide critical resources to the broader research community^34–36^. With that said, MAGs from the GEM catalog, like other MAGs generated to date, have several limitations for users to be aware of, including undetected contamination, low contiguity and incompleteness. Although these MAGs are important placeholders for many new candidate species, we expect many will be replaced in the future by higher quality MAGs or ultimately by genome sequences from clonal isolates. As we have illustrated with the large repertoire of new secondary metabolite BGCs and putative virus–host connections, we anticipate that the GEM catalog will become a valuable resource for future metabolic and genome-centric data mining and experimental validation.

## Methods

### Metagenomic samples and assembly

For genome binning, we used 10,450 metagenomic assemblies from the IMG/M database^42^ that correspond to 527 studies and 10,331 samples from a myriad of microbial environments (Supplementary Table 1). The majority (6,380 of 10,450; 61%) of metagenomes were reassembled for this work using the latest state-of-the-art assembly pipeline: read filtering with BFC, followed by assembly with metaSPAdes with the option ‘--meta’. Assembled metagenomes from IMG/M were generated using a variety of quality-control and assembly methods, as described by Huntemann et al.^62^. Where unassembled metagenomes were available, reads were mapped back to assembled contigs using BWA-MEM^63^ with default parameters, and contig coverage information was generated using SAMtools^64^.

### Metagenome binning and quality control

MAGs were recovered for the individual metagenomic assemblies using MetaBAT^65^ on the basis of tetranucleotide frequencies using v0.32.4 and v0.32.5 with option ‘--superspecific’ (Supplementary Table 2). Depth information was used when available, and contigs shorter than 3,000 bp were discarded. The resulting MAGs were refined in two stages. First, RefineM (v0.0.20)^10^ was used to remove contigs with aberrant read depth, GC content and/or tetranucleotide frequencies. Second, contigs were removed with conflicting phylum-level taxonomy. Taxonomic annotations of contigs were obtained based on protein-level alignments against the IMG/M database (downloaded 07 December 2017) using the Last aligner (v876)^66^ and taking the lowest common ancestor of taxonomically classified genes.

The completeness and contamination of all MAGs was estimated using CheckM (v1.0.11)^67^ via the lineage-specific workflow. Based on these results, we selected 52,515 MAGs that were estimated to be at least 50% complete, with less than 5% contamination and had a quality score of >50 (defined as the estimated completeness of a genome minus five times its estimated contamination). As additional indicators of completeness, we identified tRNA genes using tRNAscan-SE (v2.0)^68^ and rRNA genes using Infernal (v1.1.2)^69^ with models from the Rfam database^70^. Based on these results, we found that 9,143 of the 52,515 MAGs were classified as high quality based on the MIMAG standard (≥90% completeness, ≤5% contamination, ≥18/20 tRNA genes and presence of 5S, 16S and 23S rRNA genes), with the remaining classified as medium quality. These 52,515 MAGs form the GEM dataset.

### Metagenomic read recruitment to MAGs and reference genomes

We selected 3,170 metagenomic samples with available sequencing reads from the Joint Genome Institute and Sequence Read Archive databases to quantify mappability (Supplementary Table 4). Up to 500,000 reads from each metagenome were aligned to a database containing 52,515 GEMs and another database containing 151,730 genomes from NCBI RefSeq (release 93)^71^. We used only 500,000 reads per metagenome, representing a median of 0.84% of reads across datasets (IQR = 0.40–1.78%), to avoid the high computational cost of aligning all reads and is in line with previous analyses^4^. Read alignment was performed using Bowtie (v2.3.2) in ‘end-to-end’ mode with the option ‘--very-sensitive’, and up to 20 alignments per read were retained^72^. After alignment, we discarded low-quality reads with an average base quality score of <30, read length of <70 bp or any ambiguous base calls. Additionally, we discarded poor alignments where the edit distance exceeded 5 per 100-bp reads (that is, <95% identity).

### Clustering MAGs into species-level OTUs

The 52,515 MAGs from the GEM dataset were clustered into 18,028 species-level OTUs on the basis of 95% genome-wide ANI (Supplementary Tables 2 and 5). ANI was estimated using MUMmer (v4.0.0)^73^ with default parameters, which computes the average DNA identity across one-to-one alignment blocks between genomes. Alignments covering <30% of either genome were discarded. We used a 30% AF threshold, as opposed to a previous study that recommends using 60% AF (ref. ^74^), to avoid the formation of spurious OTUs that can result from incomplete genomes^6^. Centroid-based clustering was performed, where the MAG with the highest CheckM quality score was designated as the centroid, and all MAGs within 95% ANI to the centroid were assigned to the same cluster. As validation, we quantified the similarity of the species-level OTUs to the GTDB taxonomy for 23,009 MAGs assigned to a known species. Both datasets represented a similar number of species (3,537 OTUs versus 3,481 from the GTDB), and MAGs tended to be assigned to the same species in both databases (adjusted Rand Index = 0.99).

### Comparing MAGs to >500,000 genomes in public databases

We compared representative genomes from the 18,028 OTUs to a large number of publicly available reference genomes. Approximately 564,467 reference genomes were obtained from a variety of sources, including IMG/M (59,047 isolates, 8,412 MAGs and 7,066 SAGs), NCBI RefSeq (release 93; 151,730 isolates), GenBank (29,127 MAGs and 1,555 SAGs) and human-associated MAGs from three recent studies (307,530)^4–6^. CheckM was applied to all references and we selected those meeting the same minimum quality criteria applied to the GEM dataset (>50% completeness, <5% contamination and a quality score of >50). This resulted in a final set of 524,046 references from IMG/M (56,884 isolates, 6,146 MAGs and 1,475 SAGs), NCBI RefSeq (release 93; 150,245 isolates), GenBank (23,162 MAGs and 717 SAGs) and human-associated MAGs from three recent studies (285,417). We first used Mash (v2.0)^75^ with a sketch size of 10,000 to find the most similar reference genome to each of the 18,028 OTUs; and second, we used MUMmer (v4.0.0) with default parameters to estimate ANI between genome pairs. Based on this analysis, we found that 12,556 OTUs (69.4% of total) failed to match any reference genome at >95% ANI over >30% of the genome. Next, we identified OTUs represented only by reference genomes. First, we assigned 364,602 reference genomes to one of the 5,472 reference OTUs from the GEM dataset based on >95% ANI over >30% of the genome. The remaining 159,444 reference genomes were clustered into 27,571 additional OTUs based on 95% ANI using MUMmer. This resulted in a final dataset of 45,599 OTUs representing all GEMs and reference genomes.

### Constructing a phylogeny of nonredundant MAGs and reference genomes

We constructed a multimarker gene tree of the 45,599 OTUs based on a subset of 30 genes from the PhyEco database^76^ that were single copied in >99% of genomes searched (Supplementary Table 8). HMMER (v3.1b2)^77^ was used to identify homologs of the marker genes in the genomes of each OTU using marker-gene-specific bit-score thresholds. To mitigate missing data in incomplete genomes, we pooled homologs across genomes from the same OTU (using a maximum of ten genomes, selected on the basis of CheckM quality) for each of the 30 marker genes. We then picked the centroid gene for each marker gene in each OTU, which represents the gene with the highest similarity to other members of the same OTU. Multiple sequence alignments of the centroids were created for each marker gene using FAMSA (v1.2.5) with default parameters^78^. Columns with >10% gaps were trimmed with trimAl (v1.4; option --gt 0.90)^79^, individual marker-gene alignments were concatenated together, and sequences with >70% gaps were removed. Concatenated multiple sequence alignments contained 4,689 columns and 43,979 sequences. FastTree (v2.1.10)^80^ was used to build an approximate maximum likelihood tree using the WAG + GAMMA models.

The phylogenetic tree was used to further cluster the 45,599 OTUs into monophyletic groups at the genus, family, order, class and phylum levels using a recently described method^30^. Briefly, the tree was rooted between the bacteria and archaea, and a subclade was extracted for each domain. OTUs were clustered into monophyletic groups with bootstrap support values of >0.7 on the basis of their RED. Rank-specific RED cutoffs were identified to maximize similarity to the GTDB taxonomy for OTUs from known clades, where similarity was measured using the adjusted mutual information statistic calculated by the ‘scikit-learn’ package in Python (v0.21.3)^81^ (Supplementary Fig. 7 and Supplementary Tables 10–12). Monophyletic clades containing only GEMs were considered newly identified lineages, including those represented by a single GEM.

### Secondary metabolism

Secondary-metabolite BGCs and regions were identified using AntiSMASH (v5.1)^51^ with default settings, ignoring contigs with lengths shorter than 5 kb. BGCs were compared to those in the NCBI nucleotide database (downloaded 07 Oct 2019) using the command ‘blastn’ within the NCBI BLAST+ package (v2.9)^82^ with an *E*-value cutoff of 1 × 10^−1^. Results were parsed to evaluate top hits, and we considered redundant clusters (that is, those seen in previous sequencing efforts) to be BGC sequences matching 80% or more of the BGC query length averaging 75% or more sequence identity against a database hit. For the purpose of counting BGC biochemistry, the 46 AntiSMASH-generated specific BGC families were categorized into one of six broader groups: ‘PKS’, ‘NRPS’, ‘terpene’, ‘RiPP’, ‘AAmodifier’ and ‘other’, based on categories suggested by the BiG-SCAPE software package^83^.

### Connecting MAGs to viruses identified from IMG/VR and VirSorter

MAGs were used to predict hosts for 81,449 viral genomes from IMG/VR^56^ using a combination of CRISPR-spacer matches and sequence similarity between viruses and MAGs. CRISPR arrays were identified on contigs longer than 10 kb in MAGs using a combination of CRT^81^ and PILER-CR^84^. To minimize spurious predictions, we dropped arrays with fewer than three spacers, those with nonconserved repeats (<97% average identity to consensus repeat) or those in MAGs containing fewer than four CRISPR-associated proteins. This resulted in identification of 567,316 CRISPR spacers longer than 25 bp in 23,851 arrays in 13,540 MAGs. Protospacers were identified by aligning spacers to 760,453 IMG/VR genomes with blastn and identifying near-perfect matches (up to one mismatch covering at least 95% of the spacer length). Additionally, MAG contigs were aligned to IMG/VR genomes with blastn to identify integrated phage sequences. An IMG/VR genome was determined to be integrated in a MAG if it aligned by >90% identity over >500 bp on a contig that was >1.5 times the length of the IMG/VR genome. Contigs that were <1.5 times the length of the IMG/VR genome were considered a ‘full viral sequence’ and were discarded due to a lack of host information and the potential for inaccurate binning (that is, binning based on the virus genome characteristics rather than the host).

To maximize the number of prophages identified in MAGs, we used VirSorter (v1.0.3)^58^ to perform de novo prediction, retaining all predictions of categories 4 and 5. To exclude possible decayed prophages, that is, integrated virus genomes which are now inactive and progressively removed from the host genome, all predictions for which 30% or more of the genes displaying a best hit to Pfam were excluded (thresholds: hmmsearch score ≥ 50 and *E* ≤ 0.001). These hits were further reduced by filtering any contig that displayed >90% DNA identity over >500 bp to any of the 81,449 previously detected viral genomes from IMG/VR.

### Detailed investigation of selected virus groups

Groups of temperate or chronic viruses for which MAG-based linkages were further investigated included the DJR capsid viruses (double-stranded DNA temperate bacteriophages and archaeoviruses), inoviruses (single-stranded DNA viruses with a chronic infection cycle) and *Microviridae* (single-stranded DNA viruses, lytic or lysogenic cycle). DJR sequences were specifically identified by searching the predicted proteins from metagenome contigs for a Hidden Markov Model built from known DJR major capsid proteins, based on the sequences from Kauffman et al.^59^. The search was computed with hmmsearch from the HMMER (v3.1b2) suite, selecting hits with a hmmsearch score ≥ 50 and an *E* ≤ 0.001. An additional 81 DJR sequences were collected which had initially been predicted by VirSorter with lower confidence (category 6). Additionally, inoviruses were identified in MAGs based on a custom approach recently developed to identify inovirus-like sequences in the same metagenome assemblies before genome binning^85^.

For DJR and *Microviridae*, phylogenies were built as follows: a multiple alignment was computed with MAFFT (v7.407)^86^ using the ‘einsi’ mode; the alignment was automatically trimmed with trimAl (v1.4.rev15) using the ‘gappyout’ option^79^; and the tree was built with IQ-TREE (v1.5.5)^87^ with 1,000 ultrafast bootstraps and automatic selection of the evolutionary model. Major capsid protein sequences were used for the DJR alignment, with references obtained from Kauffman et al.^59^. Similarly, major capsid protein sequences were used for the *Microviridae* alignment, with references obtained from *Microviridae* genomes available in the NCBI RefSeq and GenBank databases (as of October 2019). In addition, the 20 best blast hits from NCBI RefSeq bacterial genomes for each GEM *Microviridae* sequence were included to incorporate additional putative prophages in the tree. For inoviruses, the gene-content-based classification previously outlined was used by mapping GEM inovirus sequences to the recently described inovirus genome catalog^85^ using the MUMmer4 function^73^ with cutoffs of 95% ANI and 70% AF.

### Reporting Summary

Further information on research design is available in the Nature Research Reporting Summary linked to this article.

## Online content

Any methods, additional references, Nature Research reporting summaries, source data, extended data, supplementary information, acknowledgements, peer review information; details of author contributions and competing interests; and statements of data and code availability are available at 10.1038/s41587-020-0718-6.

### Supplementary information

Supplementary Information Supplementary Text, Figs. 1–13 and References

Reporting Summary

Supplementary Tables Supplementary Tables 1–13 and 15–18.

Supplementary Table 14 Genome-scale metabolic models in KBase.

## Data Availability

All available metagenomic data, bins and annotations are available through the IMG/M portal (https://img.jgi.doe.gov/). Bulk download for the 52,515 MAGs is available at https://genome.jgi.doe.gov/GEMs and https://portal.nersc.gov/GEM. Genome-scale metabolic models for the nonredundant, high-quality GEMs are summarized at 10.25982/53247.64/1670777 and available in KBase (https://narrative.kbase.us/#org/jgimags). IMG/M identifiers of all metagenomes binned, including detailed information for each metagenome, are available in Supplementary Table 1.

## Code availability

The pipeline used to generate the metagenome bins is available at https://bitbucket.org/berkeleylab/metabat/src/master/.
