# Author Correction: A genomic catalog of Earth’s microbiomes

**DOI:** 10.1038/s41587-021-00898-4

**Published:** 2021-04-01

**Authors:** Stephen Nayfach, Simon Roux, Rekha Seshadri, Daniel Udwary, Neha Varghese, Frederik Schulz, Dongying Wu, David Paez-Espino, I-Min Chen, Marcel Huntemann, Krishna Palaniappan, Joshua Ladau, Supratim Mukherjee, T. B. K. Reddy, Torben Nielsen, Edward Kirton, José P. Faria, Janaka N. Edirisinghe, Christopher S. Henry, Sean P. Jungbluth, Dylan Chivian, Paramvir Dehal, Elisha M. Wood-Charlson, Adam P. Arkin, Susannah G. Tringe, Axel Visel, IMG/M Data Consortium, Helena Abreu, Silvia G. Acinas, Eric Allen, Michelle A. Allen, Lauren V. Alteio, Gary Andersen, Alexandre M. Anesio, Graeme Attwood, Viridiana Avila-Magaña, Yacine Badis, Jake Bailey, Brett Baker, Petr Baldrian, Hazel A. Barton, David A. C. Beck, Eric D. Becraft, Harry R. Beller, J. Michael Beman, Rizlan Bernier-Latmani, Timothy D. Berry, Anthony Bertagnolli, Stefan Bertilsson, Jennifer M. Bhatnagar, Jordan T. Bird, Jeffrey L. Blanchard, Sara E. Blumer-Schuette, Brendan Bohannan, Mikayla A. Borton, Allyson Brady, Susan H. Brawley, Juliet Brodie, Steven Brown, Jennifer R. Brum, Andreas Brune, Donald A. Bryant, Alison Buchan, Daniel H. Buckley, Joy Buongiorno, Hinsby Cadillo-Quiroz, Sean M. Caffrey, Ashley N. Campbell, Barbara Campbell, Stephanie Carr, JoLynn Carroll, S. Craig Cary, Anna M. Cates, Rose Ann Cattolico, Ricardo Cavicchioli, Ludmila Chistoserdova, Maureen L. Coleman, Philippe Constant, Jonathan M. Conway, Walter P. Mac Cormack, Sean Crowe, Byron Crump, Cameron Currie, Rebecca Daly, Kristen M. DeAngelis, Vincent Denef, Stuart E. Denman, Adey Desta, Hebe Dionisi, Jeremy Dodsworth, Nina Dombrowski, Timothy Donohue, Mark Dopson, Timothy Driscoll, Peter Dunfield, Christopher L. Dupont, Katherine A. Dynarski, Virginia Edgcomb, Elizabeth A. Edwards, Mostafa S. Elshahed, Israel Figueroa, Beverly Flood, Nathaniel Fortney, Caroline S. Fortunato, Christopher Francis, Claire M. M. Gachon, Sarahi L. Garcia, Maria C. Gazitua, Terry Gentry, Lena Gerwick, Javad Gharechahi, Peter Girguis, John Gladden, Mary Gradoville, Stephen E. Grasby, Kelly Gravuer, Christen L. Grettenberger, Robert J. Gruninger, Jiarong Guo, Mussie Y. Habteselassie, Steven J. Hallam, Roland Hatzenpichler, Bela Hausmann, Terry C. Hazen, Brian Hedlund, Cynthia Henny, Lydie Herfort, Maria Hernandez, Olivia S. Hershey, Matthias Hess, Emily B. Hollister, Laura A. Hug, Dana Hunt, Janet Jansson, Jessica Jarett, Vitaly V. Kadnikov, Charlene Kelly, Robert Kelly, William Kelly, Cheryl A. Kerfeld, Jeff Kimbrel, Jonathan L. Klassen, Konstantinos T. Konstantinidis, Laura L. Lee, Wen-Jun Li, Andrew J. Loder, Alexander Loy, Mariana Lozada, Barbara MacGregor, Cara Magnabosco, Aline Maria da Silva, R. Michael McKay, Katherine McMahon, Chris S. McSweeney, Mónica Medina, Laura Meredith, Jessica Mizzi, Thomas Mock, Lily Momper, Mary Ann Moran, Connor Morgan-Lang, Duane Moser, Gerard Muyzer, David Myrold, Maisie Nash, Camilla L. Nesbø, Anthony P. Neumann, Rebecca B. Neumann, Daniel Noguera, Trent Northen, Jeanette Norton, Brent Nowinski, Klaus Nüsslein, Michelle A. O’Malley, Rafael S. Oliveira, Valeria Maia de Oliveira, Tullis Onstott, Jay Osvatic, Yang Ouyang, Maria Pachiadaki, Jacob Parnell, Laila P. Partida-Martinez, Kabir G. Peay, Dale Pelletier, Xuefeng Peng, Michael Pester, Jennifer Pett-Ridge, Sari Peura, Petra Pjevac, Alvaro M. Plominsky, Anja Poehlein, Phillip B. Pope, Nikolai Ravin, Molly C. Redmond, Rebecca Reiss, Virginia Rich, Christian Rinke, Jorge L. Mazza Rodrigues, William Rodriguez-Reillo, Karen Rossmassler, Joshua Sackett, Ghasem Hosseini Salekdeh, Scott Saleska, Matthew Scarborough, Daniel Schachtman, Christopher W. Schadt, Matthew Schrenk, Alexander Sczyrba, Aditi Sengupta, Joao C. Setubal, Ashley Shade, Christine Sharp, David H. Sherman, Olga V. Shubenkova, Isabel Natalia Sierra-Garcia, Rachel Simister, Holly Simon, Sara Sjöling, Joan Slonczewski, Rafael Soares Correa de Souza, John R. Spear, James C. Stegen, Ramunas Stepanauskas, Frank Stewart, Garret Suen, Matthew Sullivan, Dawn Sumner, Brandon K. Swan, Wesley Swingley, Jonathan Tarn, Gordon T. Taylor, Hanno Teeling, Memory Tekere, Andreas Teske, Torsten Thomas, Cameron Thrash, James Tiedje, Claire S. Ting, Benjamin Tully, Gene Tyson, Osvlado Ulloa, David L. Valentine, Marc W. Van Goethem, Jean VanderGheynst, Tobin J. Verbeke, John Vollmers, Aurèle Vuillemin, Nicholas B. Waldo, David A. Walsh, Bart C. Weimer, Thea Whitman, Paul van der Wielen, Michael Wilkins, Timothy J. Williams, Ben Woodcroft, Jamie Woolet, Kelly Wrighton, Jun Ye, Erica B. Young, Noha H. Youssef, Feiqiao Brian Yu, Tamara I. Zemskaya, Ryan Ziels, Tanja Woyke, Nigel J. Mouncey, Natalia N. Ivanova, Nikos C. Kyrpides, Emiley A. Eloe-Fadrosh

**Affiliations:** 1 DOE Joint Genome Institute, Berkeley, CA USA; 2 Argonne National Laboratory, Argonne, IL USA; 3 Lawrence Berkeley National Laboratory, Berkeley, CA USA; 4 Present Address: Lawrence Berkeley National Laboratory, Berkeley, CA USA; 5 Travessa Alexandre da Conceicao, ALGAplus, Ilhavo, Portugal; 6 Department of Marine Biology and Oceanography, Institute of Marine Sciences-CSIC, Barcelona, Spain; 7 Scripps Institution of Oceanography, University of California, San Diego, La Jolla, CA USA; 8 School of Biotechnology and Biomolecular Sciences, UNSW Sydney, Sydney, New South Wales Australia; 9 Centre for Microbiology and Environmental Systems Science, University of Vienna, Vienna, Austria; 10 Department of Environmental Science, Aarhus University, Roskilde, Denmark; 11 Rumen Microbiology, Animal Science, AgResearch, Grasslands Research Centre, Palmerston North, New Zealand; 12 Department of Biology, Pennsylvania State University, University Park, Pennsylvania, PA USA; 13 Scottish Association for Marine Science, Oban, UK; 14 Department of Earth and Environmental Sciences, University of Minnesota, Minneapolis, MN USA; 15 Department of Marine Science, University of Texas Austin, Austin, TX USA; 16 Institute of Microbiology of the Czech Academy of Sciences, Praha 4, Czech Republic; 17 Department of Biology, University of Akron, Akron, OH USA; 18 Department of Chemical Engineering & eScience Institute, University of Washington, Seattle, WA USA; 19 Department of Biology, University of North Alabama, Florence, AL USA; 20 Life and Environmental Sciences and Sierra Nevada Research Institute, University of California, Merced, Merced, CA USA; 21 Ecole Polytechnique Federale de Lausanne, Lausanne, Switzerland; 22 Department of Soil Science, University of Wisconsin-Madison, Madison, WI USA; 23 School of Biological Sciences, Georgia Institute of Technology, Atlanta, GA USA; 24 Department of Aquatic Sciences and Assessment, Swedish University of Agricultural Sciences, Uppsala, Sweden; 25 Department of Biology, Boston University, Boston, MA USA; 26 University of Arkansas for Medical Sciences, Little Rock, AR USA; 27 Biology Department, University of Massachusetts Amherst, Amherst, MA USA; 28 Department of Biological Sciences, Oakland University, Rochester, MI USA; 29 Institute of Ecology and Evolution, University of Oregon, Eugene, OR USA; 30 Department of Soil and Crop Sciences, Colorado State University, Fort Collins, CO USA; 31 School of Geography and Earth Sciences, McMaster University, Hamilton, Ontario Canada; 32 School of Marine Sciences, University of Maine, Orono, ME USA; 33 Department of Life Sciences, Natural History Museum, London, UK; 34 LanzaTech., Skokie, IL USA; 35 Department of Oceanography and Coastal Sciences, Louisiana State University, Baton Rouge, LA USA; 36 Max Planck Institute for Terrestrial Microbiology, Marburg, Germany; 37 Deptartment of Biochemistry and Molecular Biology, The Pennsylvania State University, University Park, PA USA; 38 Department of Microbiology, University of Tennessee, Knoxville, TN USA; 39 Cornell University, Ithaca, NY USA; 40 Division of Natural Sciences, Maryville College, Maryville, TN USA; 41 School of Life Sciences, Arizona State University, Tempe, AZ USA; 42 University of Toronto, Toronto, Ontario Canada; 43 Lawrence Livermore National Laboratory, Livermore, CA USA; 44 Department of Biological Sciences, Clemson University, Clemson, SC USA; 45 Biology Department, Hartwick College, Oneonta, NY USA; 46 Akvaplan-niva, Fram—High North Research Centre for Climate and the Environment, Tromsø, Norway; 47 School of Science, University of Waikato, Hamilton, New Zealand; 48 Department of Soil, Water and Climate, University of Minnesota, Minneapolis, MN USA; 49 Biology Department, University of Washington, Seattle, WA USA; 50 Department of Chemical Engineering, University of Washington, Seattle, WA USA; 51 Department of the Geophysical Sciences, University of Chicago, Chicago, IL USA; 52 INRS-Centre Armand-Frappier Santé Biotechnologie, Laval, Quebec Canada; 53 Department of Chemical and Biomolecular Engineering, North Carolina State University, Raleigh, NC USA; 54 Environmental Microbiology Department, Instituto Antartico Argentino and Universidad de Buenos Aires, Buenos Aires, Argentina; 55 University of British Columbia, Vancouver, British Columbia Canada; 56 College of Earth, Ocean, and Atmospheric Sciences, Oregon State University, Corvallis, OR USA; 57 Department of Bacteriology, University of Wisconsin-Madison, Madison, WI USA; 58 Department of Microbiology, University of Massachusetts Amherst, Amherst, MA USA; 59 Department of Ecology and Evolutionary Biology, University of Michigan, Ann Arbor, MI USA; 60 Commonwealth Scientific Industrial Research Organisation, Brisbane, Queensland Australia; 61 College of Natural and Computational Sciences, Addis Ababa University, Addis Ababa, Ethiopia; 62 Laboratorio de Microbiología Ambiental, Centro para el Estudio de los Sistemas Marinos (CESIMAR, CONICET), Puerto Madryn, Argentina; 63 California State University, San Bernadino, San Bernardino, CA USA; 64 Department of Marine Microbiology and Biogeochemistry, NIOZ, Royal Netherlands Institute for Sea Research and Utrecht University, AB Den Burg, the Netherlands; 65 Department of Bacteriology, Wisconsin Energy Institute, University of Wisconsin-Madison, Madison, WI USA; 66 Centre for Ecology and Evolution in Microbial Model Systems (EEMiS), Linnaeus University, Växjö, Sweden; 67 Department of Biology, West Virginia University, Morgantown, WV USA; 68 Department of Biological Sciences, University of Calgary, Calgary, Alberta Canada; 69 J. Craig Venter Institute, La Jolla, CA USA; 70 Department of Ecosystem and Conservation Sciences, University of Montana, Missoula, MT USA; 71 Department of Geology and Geophysics, Woods Hole Oceanographic Institution, Woods Hole, MA USA; 72 Departments of Chemical Engineering and Applied Chemistry and Cell and Systems BIology, University of Toronto, Toronto, Ontario Canada; 73 Department of Microbiology and Molecular Genetics, Oklahoma State University, Stillwater, OK USA; 74 Visolis, Hayward, CA USA; 75 Great Lakes Bioenergy Research Center, University of Wisconsin-Madison, Madison, WI USA; 76 Department of Biology, Widener University, Chester, PA USA; 77 Department of Earth System Science, Stanford University, Stanford, CA USA; 78 Department of Ecology, Environment and Plant Sciences, Science for Life Laboratory, Stockholm University, Stockholm, Sweden; 79 Departments of Microbiology, The Ohio State University, Columbus, OH USA; 80 Texas A&M University, College Station, TX USA; 81 Human Genetics Research Center, Baqiyatallah University of Medical Sciences, Tehran, Iran; 82 Organismic and Evolutionary Biology, Harvard University, Cambridge, MA USA; 83 Department of Biomass Science and Conversion Technology, Sandia National Laboratory, Livermore, CA USA; 84 Ocean Sciences Department, University of California, Santa Cruz, Santa Cruz, CA USA; 85 Natural Resources Canada, Geological Survey of Canada, Calgary, Alberta Canada; 86 Graduate Group in Ecology, University of California, Davis, Davis, CA USA; 87 Department of Earth and Planetary Sciences, University of California, Davis, Davis, CA USA; 88 Lethbridge Research and Development Centre, Agriculture and Agri-Food Canada, Lethbridge, Alberta Canada; 89 Center for Microbial Ecology, Michigan State University, East Lansing, MI USA; 90 Department of Crop and Soil Sciences, University of Georgia Griffin Campus, Griffin, GA USA; 91 Department of Microbiology and Immunology, University of British Columbia, Vancouver, British Columbia Canada; 92 Department of Chemistry and Biochemistry, Thermal Biology Institute, and Center for Biofilm Engineering, Montana State University, Bozeman, MT USA; 93 Centre for Microbiology and Environmental Systems Science, Department of Microbiology and Ecosystem Science, Division of Microbial Ecology, University of Vienna, Vienna, Austria; 94 Department of Civil and Environmental Engineering, University of Tennessee, Knoxville, TN USA; 95 School of Life Sciences, University of Nevada, Las Vegas, Las Vegas, NV USA; 96 Research Center for Limnology (LIPI), Indonesian Institute of Sciences, Division of Inland Waterways Dynamics, Cibinong-Bogor, Indonesia; 97 Center for Coastal Margin Observation & Prediction (CMOP), Oregon Health & Science University, Portland, OR USA; 98 Biotechnological Management of Resources Network, Institute of Ecology, Xalapa, Mexico; 99 Department of Animal Science, University of California Davis, Davis, CA USA; 100 Diversigen, Houston, TX USA; 101 Department of Biology, University of Waterloo, Waterloo, Ontario Canada; 102 Duke University Marine Laboratory, Beaufort, NC USA; 103 Biological Sciences Division, Earth and Biological Sciences Directorate, Pacific Northwest National Laboratory, Richland, WA USA; 104 AnimalBiome, Oakland, CA USA; 105 Institute of Bioengineering, Research Center of Biotechnology, Russian Academy of Sciences, Moscow, Russia; 106 Division of Forestry and Natural Resources, West Virginia University, Morgantown, WV USA; 107 Department of Chemical and Biomolecular Engineering, North Carolina State University, Raleigh, NC USA; 108 Donvis Ltd, Ashhurst, New Zealand; 109 Department of Molecular and Cell Biology, University of Connecticut, Mansfield, CT USA; 110 School of Civil and Environmental Engineering, Georgia Institute of Technology, Atlanta, GA USA; 111 School of Life Sciences, Sun Yat-sen University, Guangzhou, China; 112 Laboratorio de Microbiología Ambiental, Instituto de Biología de Organismos Marinos, Puerto Madryn, Argentina; 113 Geological Institute, Department of Earth Sciences, ETH Zürich, Zürich, Switzerland; 114 Departamento de Bioquímica, Instituto de Química, Universidade de São Paulo, São Paulo, Brazil; 115 Great Lakes Institute for Environmental Research, University of Windsor, Windsor, Ontario Canada; 116 Departments of Civil and Environmental Engineering, and Bacteriology, University of Wisconsin-Madison, Madison, WI USA; 117 Commonwealth and Scientific Industrial Research Organisation, Brisbane, Queensland Australia; 118 School of Natural Resources and the Environment, University of Arizona, Tucson, AZ USA; 119 School of Environmental Sciences, University of East Anglia, Norwich Research Park, Norwich, UK; 120 Exponent Consulting, Pasadena, CA USA; 121 Department of Marine Sciences, University of Georgia, Athens, GA USA; 122 Division of Hydrologic Sciences, Desert Research Institute, Reno, NV USA; 123 Microbial Systems Ecology, Department of Freshwater and Marine Ecology, Institute for Biodiversity and Ecosystem Dynamics, University of Amsterdam, Amsterdam, the Netherlands; 124 Oregon State University, Corvallis, OR USA; 125 School of Geographical Sciences, University of Bristol, Bristol, UK; 126 Departments of Chemical Engineering and Applied Chemistry and Cell and Systems BIology, University of Toronto, Toronto, Ontario Canada; 127 Department of Civil & Environmental Engineering, University of Washington, Seattle, WA USA; 128 Department of Plants, Soils and Climate, Utah State University, Logan, UT USA; 129 Department of Chemical Engineering, University of California, Santa Barbara, Santa Barbara, CA USA; 130 Department of Plant Biology, University of Campinas, Campinas, Brazil; 131 Microbial Resources Division, Research Center for Chemistry, Biology and Agriculture, University of Campinas, Campinas, Brazil; 132 Department of Geosciences, Princeton University, Princeton, NJ USA; 133 Institute for Environmental Genomics, University of Oklahoma, Norman, OK USA; 134 Department of Biology, Woods Hole Oceanographic Institution, Woods Hole, MA USA; 135 Novozymes, Durham, NC USA; 136 Centro de Investigacion y de Estudios Avanzados del IPN (CINVESTAV), Unidad Irapuato, Irapuato, Mexico; 137 Department of Biology, Stanford University, Stanford, CA USA; 138 Oak Ridge National Laboratory, Oak Ridge, TN USA; 139 Department of Microorganisms, Leibniz Institute DSMZ - German Collection of Microorganisms and Cell Cultures, Braunschweig, Germany; 140 Department of Forest Mycology and Plant Pathology, Science for Life Laboratory, Swedish University of Agricultural Sciences, Uppsala, Sweden; 141 Genomic and Applied Microbiology & Göttingen Genomics Laboratory, Institute of Microbiology and Genetics, Georg-August University of Göttingen, Göttingen, Germany; 142 Faculty of Biosciences, Norwegian University of Life Sciences, Ås, Norway; 143 Department of Biological Sciences, University of North Carolina Charlotte, Charlotte, NC USA; 144 Department of Biology, New Mexico Institute of Mining and Technology, Socorro, NM USA; 145 Microbiology Department, and Byrd Polar and Climate Research Center, The Ohio State University, Columbus, OH USA; 146 Australian Centre for Ecogenomics/School of Chemistry and Molecular Biosciences, University of Queensland, Brisbane, Queensland Australia; 147 Research Computing Division, Harvard Medical School, Boston, MA USA; 148 Division of Pulmonary Sciences and Critical Care Medicine, University of Colorado Denver, Anschutz Medical Campus, Aurora, CO USA; 149 Department of Biological Sciences, University of Cincinnati, Cincinnati, OH USA; 150 Department of Systems Biology, Agricultural Biotechnology Research Institute of Iran, Agricultural Research, Education, and Extension Organization, Karaj, Iran; 151 Department of Ecology and Evolutionary Biology, University of Arizona, Tucson, AZ USA; 152 Department of Civil and Environmental Engineering, University of Vermont, Burlington, VT USA; 153 University of Nebraska - Lincoln, Lincoln, NE USA; 154 Department of Microbiology and Molecular Genetics, Michigan State University, East Lansing, MI USA; 155 Faculty of Technology and Centrum for Biotechnology, Bielefeld University, Bielefeld, Germany; 156 California Lutheran University, Thousand Oaks, CA USA; 157 Departamento de Bioquímica, Instituto de Química, Universidade de São Paulo, São Paulo, Brazil; 158 Faculty of Science, University of Calgary, Calgary, Alberta Canada; 159 Life Sciences Institute, Deparment of Medicinal Chemistry, University of Michigan, Ann Arbor, MI USA; 160 Limnological Institute, Siberian Branch of the Russian Academy of Sciences, Irkutsk, Russia; 161 Department of Environmental Science, School of Natural Sciences, Technology and Environmental Studies, Södertörn University, Huddinge Municipality, Huddinge, Sweden; 162 Department of Biology, Kenyon College, Gambier, OH USA; 163 Genomics for Climate Change Research Center, University of Campinas, Campinas, Brazil; 164 Department of Civil and Environmental Engineering, Colorado School of Mines, Golden, CO USA; 165 Bigelow Laboratory for Ocean Sciences, East Boothbay, ME USA; 166 Departments of Microbiology and Civil, Environmental, and Geodetic Engineering, The Ohio State University, Columbus, OH USA; 167 National Biodefense Analysis and Countermeasures Center, Frederick, MD USA; 168 Department of Biological Sciences, Northern Illinois University, DeKalb, IL USA; 169 Department of Earth Science and Marine Science Institute, University of California, Santa Barbara, Santa Barbara, CA USA; 170 School of Marine & Atmospheric Sciences, Stony Brook University, Stony Brook, NY USA; 171 Department of Molecular Ecology, Max Planck Institute for Marine Microbiology, Bremen, Germany; 172 Department of Environmental Science, University of South Africa, Pretoria, South Africa; 173 Deptartment of Marine Sciences, University of North Carolina at Chapel Hill, Chapel Hill, NC USA; 174 Centre for Marine Science and Innovation & School of Biological, Earth and Environmental Sciences, University of New South Wales, Sydney, New South Wales Australia; 175 Department of Biological Sciences, University of Southern California, Los Angeles, Los Angeles, CA USA; 176 Department of Plant, Soil and Microbial Sciences, Michigan State University, East Lansing, MI USA; 177 Department of Biology, Williams College, Williamstown, MA USA; 178 University of Southern California, Los Angeles, Los Angeles, CA USA; 179 School of Chemistry and Molecular Biosciences, University of Queensland, Brisbane, Queensland Australia; 180 Departamento de Oceanografía & Instituto Milenio de Ocenografía, Universidad de Concepción, Bio Bio, Chile; 181 Department of Bioengineering, University of Massachusetts Dartmouth, Dartmouth, MA USA; 182 IBG-5, Karlsruhe Institute of Technology, Karlsruhe, Germany; 183 Ludwig-Maximilians-Universität München, Munich, Germany; 184 Department of Biology, Concordia University, Montreal, Quebec Canada; 185 School of Veterinary Medicine, Population Health and Reproduction, University of California, Davis, Davis, CA USA; 186 KWR Water Research Institute, Nieuwegein, the Netherlands; 187 Department of Biological Sciences, University of Wisconsin-Milwaukee, Milwaukee, WI USA; 188 Chan Zuckerberg Biohub, Stanford, CA USA; 189 Department of Civil Engineering, University of British Columbia, Vancouver, British Columbia Canada

**Keywords:** Computational biology and bioinformatics, Microbiology

Correction to: *Nature Biotechnology* 10.1038/s41587-020-0718-6, published online 9 November 2020.

In the version of this article initially published, four people were missing from the alphabetical list of IMG/M Data Consortium members: Lauren V. Alteio of the Centre for Microbiology and Environmental Systems Science, University of Vienna, Vienna, Austria; Jeffrey L. Blanchard of the Biology Department, University of Massachusetts Amherst, Amherst, MA, USA; Kristen M. DeAngelis of the Department of Microbiology, University of Massachusetts Amherst, Amherst, MA, USA; and William Rodriguez-Reillo of the Research Computing Division, Harvard Medical School, Boston, MA, USA. The error has been corrected in the PDF and HTML versions of the article.

