# Supplementary material for: A genomic catalog of Earth’s microbiomes
Source: Nat Biotechnol. 2020 Nov 9;39(4):499–509. doi: 10.1038/s41587-020-0718-6 (PMC8041624; doi:10.1038/s41587-020-0718-6)
Supplement: Supplementary file 1 — Supplementary Text, Figs. 1–13 and References [file 41587_2020_718_MOESM1_ESM.pdf]

nature portfolio

https://doi.org/10.1038/s41587-020-0718-6

Supplementary information

A genomic catalog of Earth's microbiomes

In the format provided by the authors and unedited

Supplementary Text

Comparison of species-level bins with MAGs from Parks et al.

We compared the MAGs from the GEM catalogue to MAGs from the UBA dataset [1] for 440 samples analyzed by both studies and represented by a single sequencing experiment in the NCBI SRA. All MAGs were clustered into species-level OTUs together with the other genomes from the GEM catalogue and reference databases. Overall there were 1,840 UBA genomes and 1,653 GEM genomes representing 1,046 and 951 species-level OTUs, respectively. Parks et al. applied some additional quality filters to the UBA genomes (N50 >10Kb and # contigs < 500), so these were also applied to the GEM catalogue for a fair comparison. Thus, all MAGs passed the same set of quality criteria and were clustered together based on 95% ANI with a 50% alignment fraction threshold into species OTUs. We found 743 OTUs in both datasets, yielding an overlap of 78% relative to the GEM catalogue and 71% relative to the UBA dataset. The remaining differences are likely due to a combination of different numbers of MAGs recovered per sample in each study, and different species-level OTUs represented by those MAGs.

Quality comparison of species-level OTUs for clusters and singletons

Of the 18,028 species-level OTUs recovered from the GEM catalogue, a large number (12,556) were found to represent new candidate species that were distinct at 95% ANI from existing reference genomes. However, a large proportion of this novelty was found to reside in singleton clusters (8,693 OTUs). To explore this issue further, we compared the quality of new OTUs versus known OTUs, as well as singleton OTUs versus non-singleton OTUs using several metrics, including: estimated genome completeness, contamination, N50, new protein clusters, and habitat distribution (Table S6). New OTUs and singleton OTUs tend to have slightly lower genome quality compared to known OTUs and non-singleton OTUs. New OTUs tend to be enriched in aquatic and terrestrial environments. The environmental distribution of singleton and non-singleton OTUs differed, which is likely explain by differences in the underlying distribution of samples and microbial diversity of those samples. To validate the novelty of new OTUs, we performed taxonomic annotation using GTDB-tk, revealing >99% were unannotated at the species rank. Additionally, we constructed a phylogenetic tree of all OTUs and clustered the OTUs into lineages at higher ranks. This phylogenetic analysis revealed a large number of new genus (5,463) and family level lineages (1,525), indicating many of the new OTUs lie well beyond the threshold for species-level delineation.

Comparison of BGCs with complete NCBI genomes

Modular BGC families, such as Type-1 modular polyketide synthases (T1PKS) [2] and non-ribosomal peptide synthetases (NRPS), are highly repetitive and notoriously difficult to assemble without long-read sequences to help bridge the module repeats [3]. For reference, we conducted a parallel analysis on NCBI genomes labeled as "complete" and found 76,107 clusters within 64,673 regions across 12,668 complete genomes, with fewer than 1% located on contig edges and averaging 6 BGCs per genome. A histogram of BGC length shows that the GEM catalogue contains similar counts for small 1-5 gene BGCs and more complex multi-gene BGCs, but fewer very large (40-100 kb) BGC systems, most of which in that size range are dominated by modular PKS and NRPS genes (Figure S11).

Horizontal transfer of BGCs in nature also likely plays a factor in the fragmentation and lower counts of BGCs in the catalogue. BGCs are well known to be horizontally transferred between bacterial and fungal species, and it is common to see G+C content and codon bias within BGCs that differ dramatically from the host genome [4], as well as integrase or viral remnants or non-coding sequence flanking BGC sequences. Indicators like this are commonly used to interpret BGC boundaries, and some species have seemingly adapted their genome structure to accommodate uptake and regulation of BGCs, resulting in genome regions classically termed "pathogenicity islands" or genomic islands [5]. BGCs are also regularly found on plasmids, another common horizontal transfer vector. Therefore, we expect that many BGC-containing contigs may have been lost or mis-assigned in the assembly and binning process of constructing the GEM catalogue, resulting in an undercount and/or increased fragmentation of BGCs, with frequent loss of pathogenicity islands and loss of plasmids.

Genome-scale metabolic models

All metabolic models were built and reconstructed using the "Build Metabolic Model" App in KBase: https://narrative.kbase.us/#catalog/apps/fba_tools/build_metabolic_model/release. GEMs and reference genomes were annotated with RAST [6] in KBase, as the ModelSEED pipeline uses RAST functional roles to map genes to biochemical reactions [7, 8] . The metabolic models were used to assess pathway presence (as defined by KEGG [9]) as detected by a complete flux pathway within the defined environments. A pathway was determined present or not detected by computing the number of gene-associated functional reactions (GAFRs) in each pathway across all models. GAFRs are defined as reactions in a model that are involved in pathways that offer uninterrupted mass-balanced routes from nutrients to biomass and byproducts. Thus, GAFRs exclude reactions that are part of fragmentary, incomplete, and likely nonfunctional pathways.

The GEMs were expected to have some gaps due to incomplete genome reconstruction, and gaps will occur due to errors and omissions in functional annotations. To address these issues, all GEMs were subjected to a gap filling operation [10] that ensured that every high-quality GEM was capable of producing biomass from a least one carbon source. Out of the 3,732 high-quality GEMs, we analyzed metabolic models for a subset of 3,270, excluding MAGs with biome labeled as "other" and biomes with low MAG counts (<40 MAGs). Out of the subset of 3,270, 15 did not successfully complete the gap filling operation, resulting in 3,255 GEMs with metabolic models.

A threshold-based approach was used to define each pathway as being either present or not detected in each GEM and reference genome analyzed. The individual thresholds were assessed by calculating the difference between average and standard deviation of GAFRs for each individual pathway. Pathways above the calculated threshold (Table S14) are considered "present" for a given model/organism. Only pathways with five or more GAFRs were considered in this study to account for smaller linear pathway definitions by KEGG. Based on this analysis, the fraction of GEMs in each environment that were determined to possess active pathways are shown in Figure S7. Three scenarios were detected: (1) pathways effectively present in all genomes in the environment (light color cells); (2)

pathways not detected in any genomes (dark color cells); and (3) pathways present in some genomes but not others. This corresponds with pathways that are likely essential, pathways that likely contribute little to fitness, and pathways that may contribute to potential co-metabolism and trophic dependency within the microbial community. Differences can also be seen in patterns of pathway presence between environments, although similar environments do cluster together (e.g., human and mammal). To validate the high-quality GEM metabolic models, pathway presence profiles were computed for reference genomes associated with humans and the built environment, as these two environments have >100 GEMs with associated reference genomes (Figure S8). The resulting profiles were nearly identical for all pathways. Pearson correlation coefficients were calculated for each GEM and corresponding reference genome across 55 metabolic pathways, with an average value >0.98. When the GEM and reference genomes were randomly paired and a Pearson correlation was calculated, the average correlation dropped to ~0.82, indicating that the high correlation previously obtained reflects the similarity of the GEM and reference genome. All data and calculations used in these analyses are available in the Table S14.

Custom species and gene trees for mcrABG-encoding Archaea

The 15 archaeal GEMs which encoded for the mcrABG operon were added to a representative set of 652 publicly available archaeal genomes downloaded from the Integrated Microbial Genomes and Microbiomes (IMG/M) online database [11] (database accessed May 2019) and the GTDB [12] . A species tree was built from 56 universal marker proteins from the COG database [13] which were identified with hmmsearch v3.1b2 [14] using a specific hidden Markov model for each of the markers. For every protein, alignments were prepared with MAFFT v 7.294b [15] and subsequently trimmed with BMGE using BLOSUM30 [16]. Genomes with less than 20 marker proteins and genomes that had more than five duplicated marker proteins were removed from the alignment. Aligned marker proteins were concatenated to a supermatrix which comprised 17,240 informative sites and a phylogenetic tree was built with IQ-TREE v1.6.12 [17] using model LG4X+F with the ultrafast bootstrap option [18]. The phylogenetic tree was visualized with iTol v5 [19].

McrA (MCR_alpha), McrB (MCR_beta), and McrG (MCR_gamma) were identified in the same set of genomes used to build the species tree of the Archaea using models from the PfamA database v29.0 [20]. Significant hits for McrABG were extracted from genomes which encoded all three genes. Sequences were aligned with MAFFT v7.294b [15], trimmed with trimal v1.4 [21] to remove positions with more than 90% of gaps (-gt 0.1). A phylogenetic tree was built from a concatenated alignment of McrABG (1,199 informative positions) with IQ-tree v1.6.12 using model LG+F+R5 chosen based on the model test feature [22]. The phylogenetic tree was visualized with iTol v5 [19].

Benchmarking host-prediction methods

We used MAGs from the GEM catalogue to predict hosts for IMG/VR viruses using a combination of CRISPR targeting and prophages (see Methods in main text for details). Each MAG was taxonomically annotated by the GTDB, enabling us to link each virus to a host at a variety of taxonomic ranks (phylum, class, order, family, genus, and species). The predicted host taxonomy for viruses was then validated using two separate approaches.

We first compared the host taxonomy of viruses determined by genome sequences matches (i.e. prophages) versus predictions based on spacer matches. For each method, and at each taxonomic rank, the predicted host was determined based on the most commonly observed taxon. These predicted hosts were then compared between methods. For viruses with hosts predicted by both methods, we observed agreement at the following ranks: phylum (91.9%), class (91.8%), order (88.5%), family (82.4%), genus (73.9%), species (53.2%). We found that viruses matching MAG contigs 'end-to-end' often had discordant predictions with CRISPR spacers. We reasoned that these contigs of entirely viral origin may be incorrectly assigned to a MAG during the binning process, likely because of differences in GC content and codon usage between viral and host genome, and/or because of differences in genome copy number (i.e. coverage) for viruses actively replicating. When only considering MAG contigs >1.5x the length of matched viruses, the agreement with CRISPR spacers was increased: phylum (96.9%), class (96.9%), order (94.9%), family (88.6%), genus (79.3%), species (56.3%). Finally, we found that agreement between methods further increased when only considering "confident" predictions by each method (>10 virus-host connections with >90% agreement within-method): phylum (99.2%), class (99.2%), order (99.0%), family (98.7%), genus (98.0%), species (98.6%).

Second, we evaluated the "purity" of predicted hosts for each virus at different taxonomic ranks. This was performed for each prediction method. Purity was defined at each taxonomic rank by (1) identifying the most common host taxon for a virus, and (2) determining the percent of predictions matching this taxon. For example, at the phylum-rank, a virus matching 9 Proteobacteria and 1 Bacteroidetes would have a purity of 90%. For hosts predicted based on CRISPR-targeting, we observed the following purity values: phylum (99.1%), class (99.1%), order (98.5%), family (95.7%), genus (91.1%), species (84.9%). For hosts predicted based on genome sequence matches, we observed the following purity values: phylum (99.6%), class (99.6%), order (99.0%), family (95.2%), genus (89.0%), species (75.8%).

Supplementary Figures

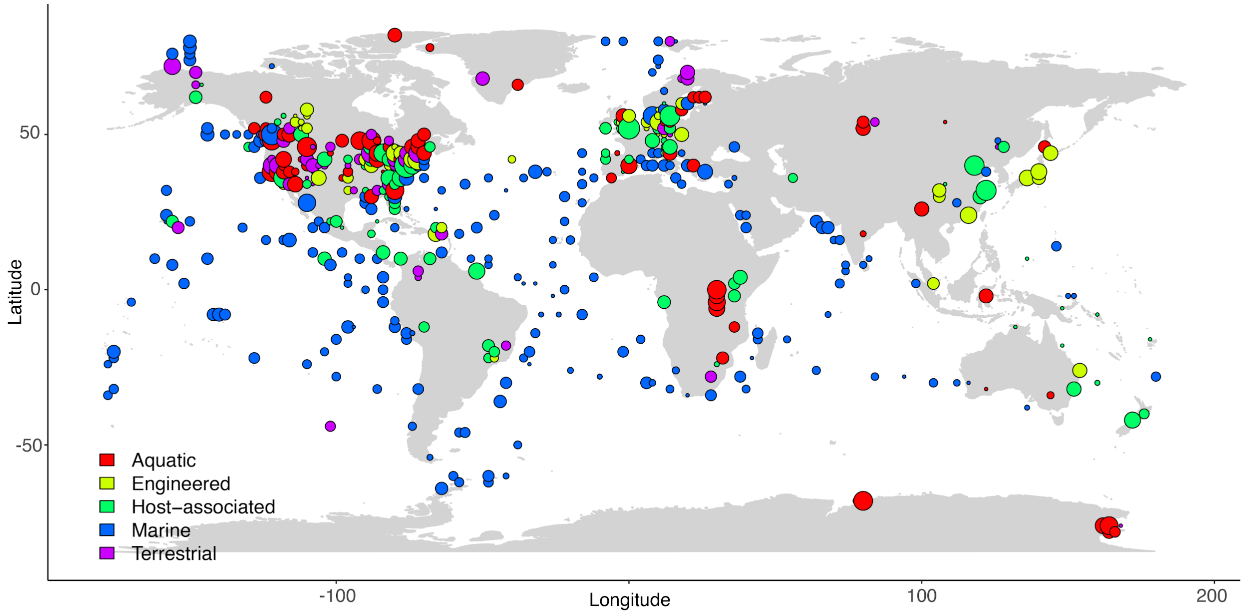

Figure S1. Global distribution of 52,515 MAGs. Each point indicates one sampling location, rounded to the nearest degree, with point size reflecting the number of MAGs on a log10 scale, and point color indicating the habitat.

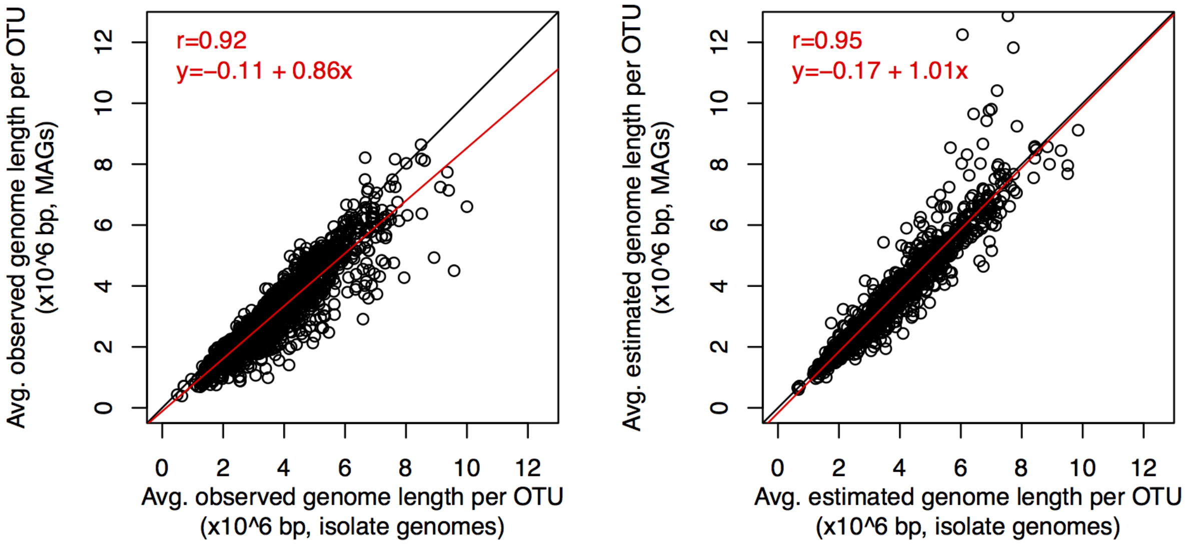

Figure S2. Comparison of conspecific MAGs and isolate genomes. MAGs from the GEM catalogue were clustered with reference genomes into OTUs at 95% ANI. Each point represents one OTU containing at least one GEM and one isolate reference genome. In both panels, the x-axis indicates the average observed genome length of isolates genomes. In the left panel, the y-axis indicates the average observed genome length of MAGs, while this is normalized for estimated genome completeness on the right. The average genome sizes of

MAGs and isolate genomes from the same species is highly correlated with a slope close to one, indicating no systematic loss or gain of gene content.

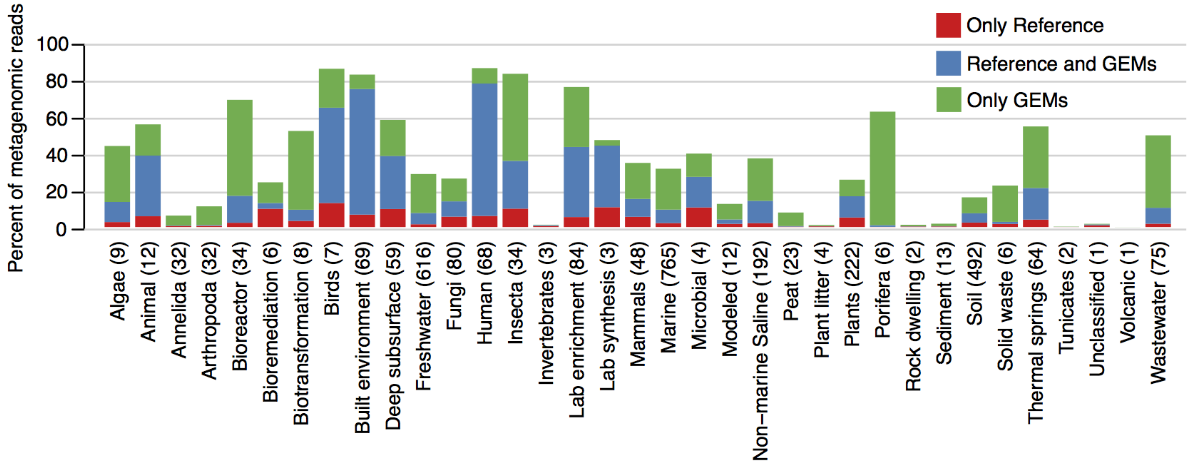

Figure S3. MAGs increase the mappability of metagenomes. Sequencing reads from 3,170 metagenomes were mapped to a database containing 52,515 MAGs from the GEM catalogue and 151,730 isolate genomes from NCBI RefSeq. The barplot shows the percent of high-quality reads mapping to genomes from each database with >95% identity. The number of metagenomes is indicated in parenthesis.

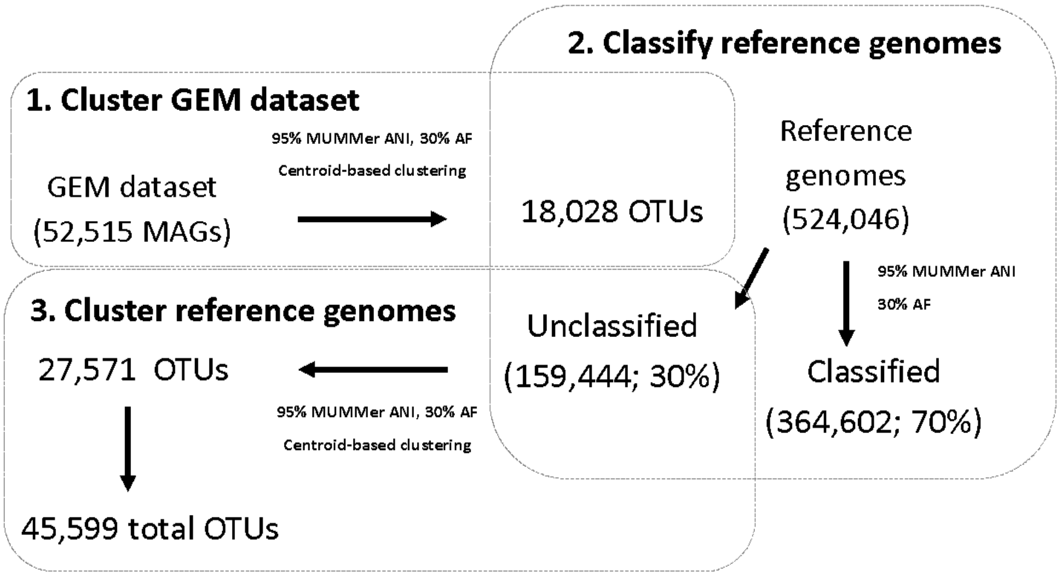

Figure S4. Workflow for clustering GEMs and reference genomes into species-level OTUs.

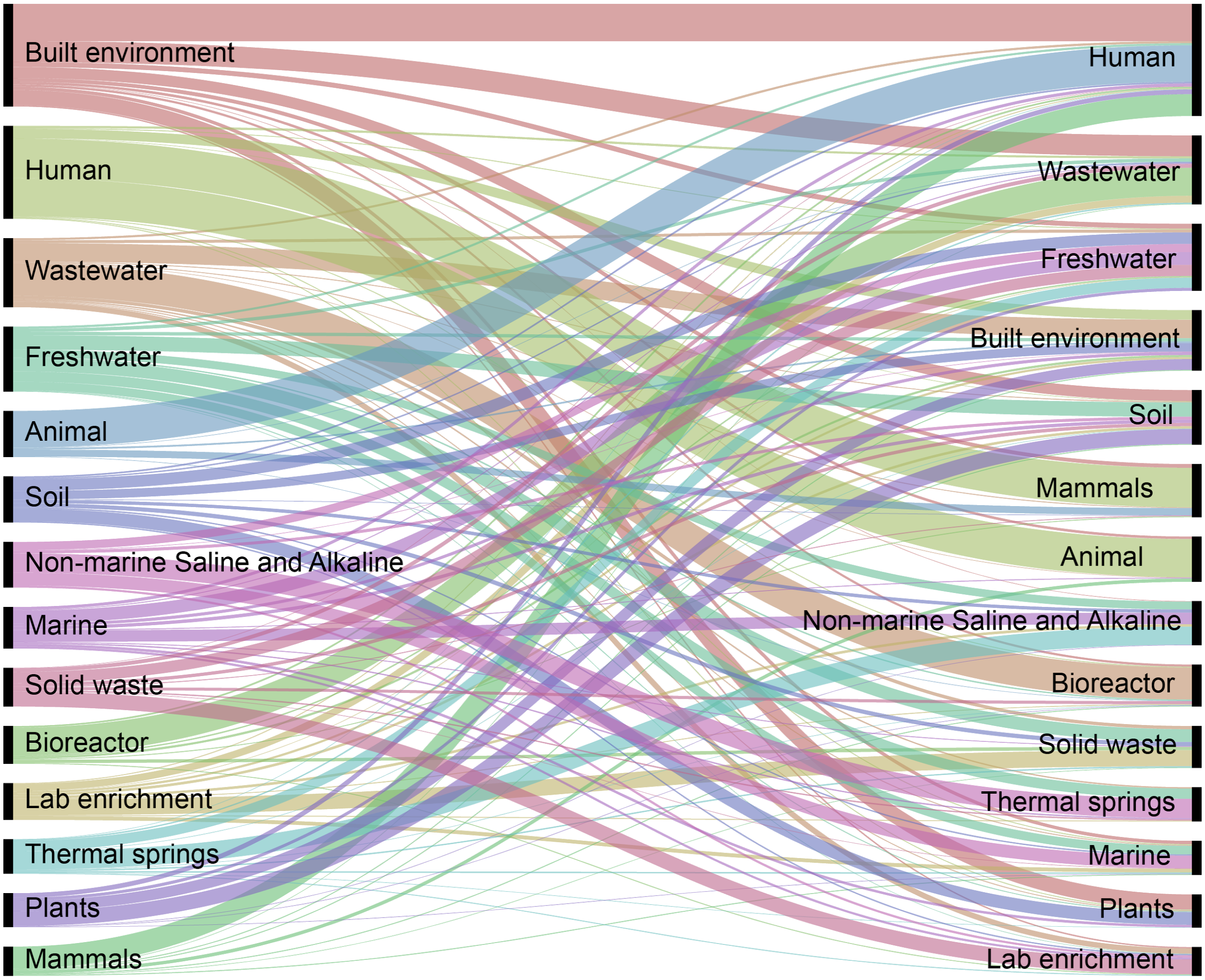

Figure S5. OTUs found across distinct biomes. Biomes are listed on the left and right with OTUs connecting them. For improved clarity, only a subset of biomes with the most connections are shown. Biomes with shared OTUs tend to be related.

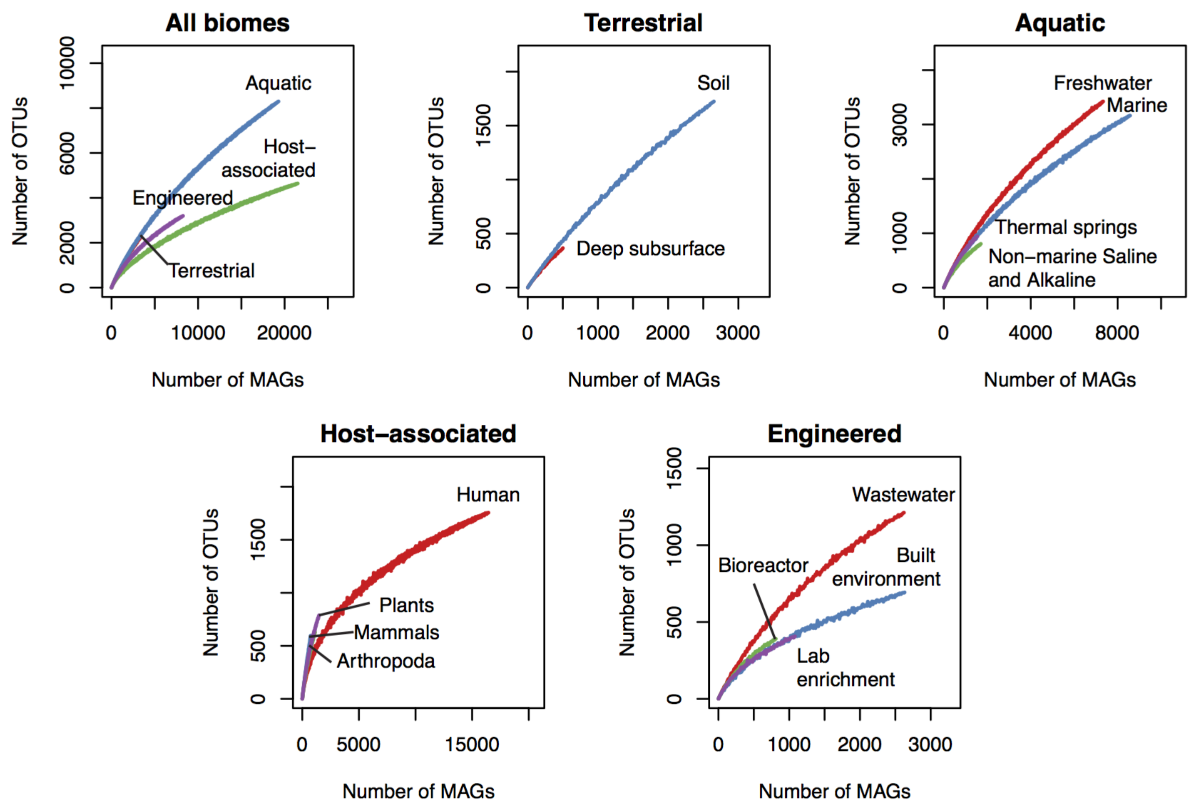

Figure S6. Accumulation of species-level OTUs across environments.

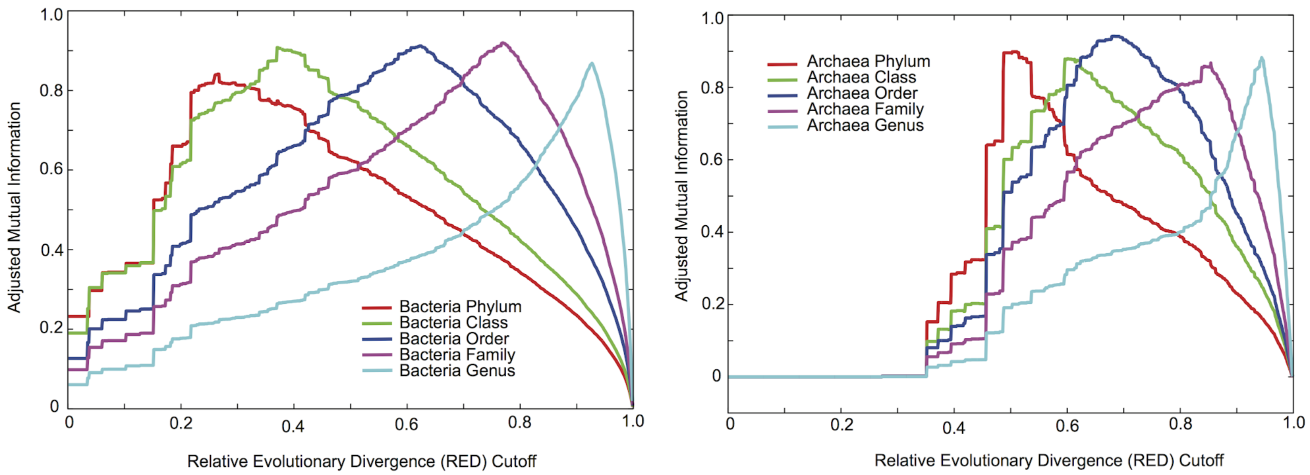

Figure S7. Identification of RED cutoffs for phylogenetic clustering.

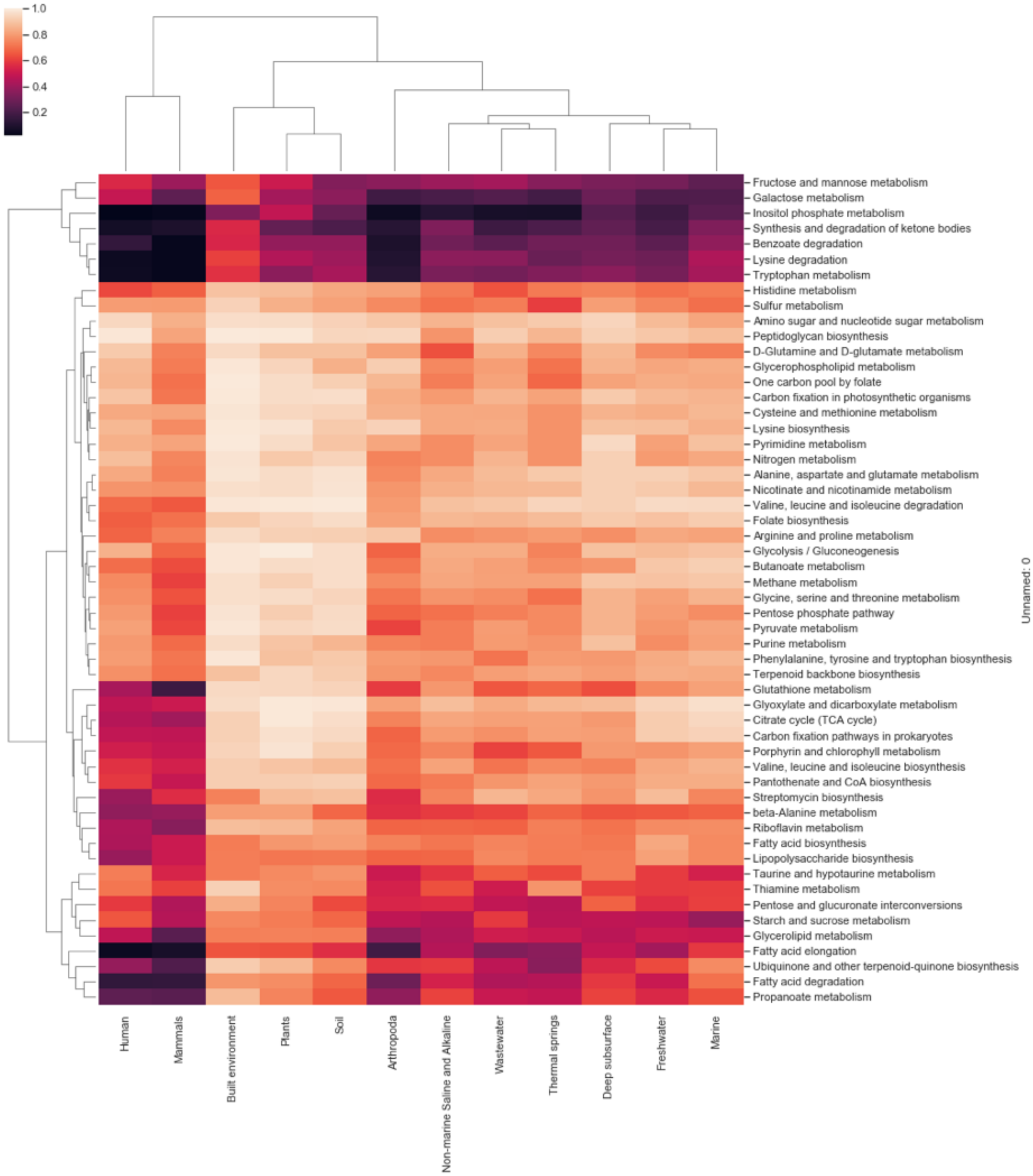

Figure S8. Hierarchically clustered heatmap of the fraction of GEMs in each environment that were determined to possess active pathways from genome-scale metabolic models. The color scheme indicates pathway presence or not detected. Light cells indicate a pathway is present in all or most all genomes in the environment. Dark cells show environments where the pathway was not detected in those genomes.

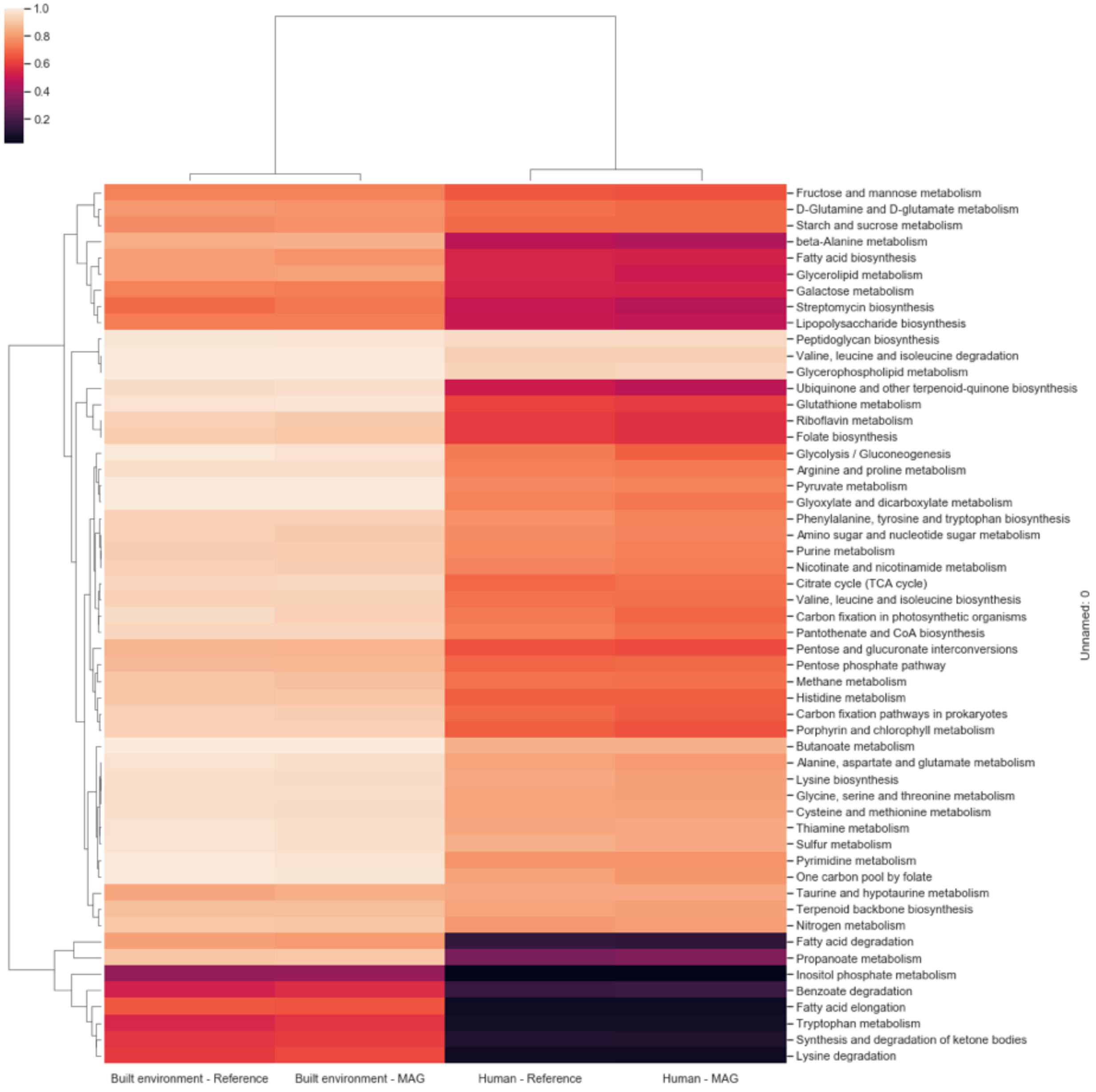

Figure S9. Hierarchically clustered heatmap of the fraction of GEMs vs close Refseq genome for the “Built environment” and “Human” biomes, that were determined to possess active pathways from genome-scale metabolic models. The color scheme indicates pathway presence or not detected. Light cells indicate a pathway is present in all or most all genomes in the environment. Dark cells show environments where the pathway was not detected in those genomes.

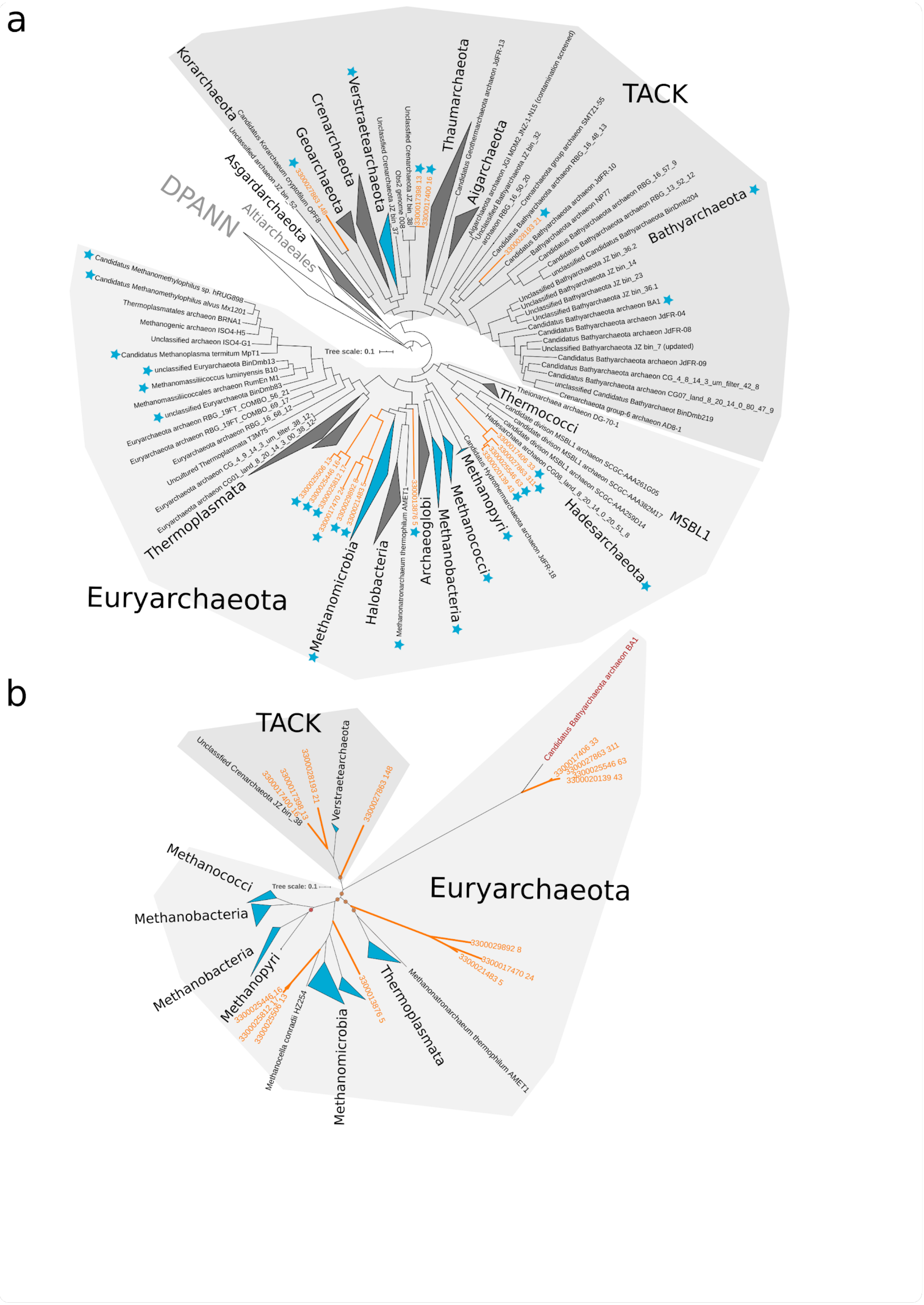
a
TACK
Korarchaeota
Verstraetearchaeota
Crenarchaeota
Geoarchaeota
Thaumarchaeota
Aigarchaeota
Bathyarchaeota
DPANN
Asgardarchaeota
Altiarchaeales
Thermococci
MSBL1
Hadesarchaeota
Methanopyri
Methanococci
Methanobacteria
Archaeoglobi
Halobacteria
Methanomicrobia
Thermoplasmata
Euryarchaeota
Tree scale: 0.1
b
TACK
Verstraetearchaeota
Euryarchaeota
Methanococci
Methanobacteria
Methanopyri
Methanomicrobia
Thermoplasmata
Tree scale: 0.1

Figure S10. Archaeal GEMs expand the diversity of methanogens in the domain Archaea. A) Maximum likelihood phylogenetic tree (IQ-tree, LG4X+F) of Archaea showing the position of archaeal GEMs (highlighted in orange) and previously published archaeal genomes which encode for the mcrABG operon (highlighted with a blue star). Clades that predominantly contain genomes that encode for the mcrABG operon are highlighted in blue and also labeled with a blue star. The tree is rooted at the DPANN supergroup. Scale bar indicates substitutions per site. B) Unrooted maximum likelihood phylogenetic tree (IQ-tree, LG4X+F) inferred from a concatenated alignment of McrABG. GEMs are highlighted in orange. Clades that predominantly contain genomes that encode for the mcrABG operon are highlighted in blue. The archaeal genome highlighted in red (a member of TACK supergroup in species tree) most likely acquired its mcrABG operon from euryarchaeotal GEMs (Hadesarchaeota in the species tree) through horizontal gene transfer. Branches with low bootstrap support (<70%) are indicated by filled red circles, branches with medium support (between 70-90%) are indicated by filled orange circles, branches without filled circles are highly supported (>90%). Scale bar indicates substitutions per site.

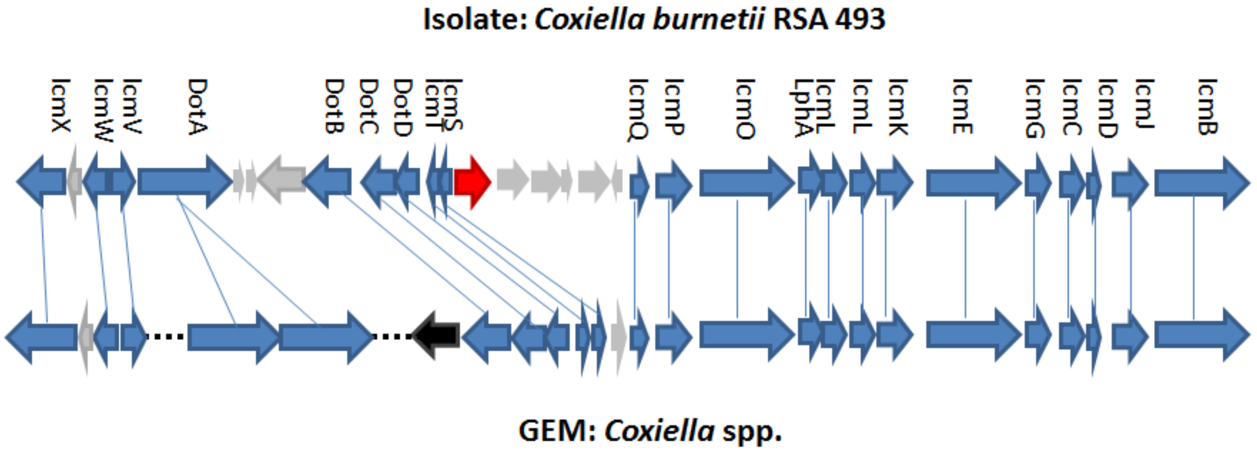

Figure S11. Schematic comparing the organization of putative T4SS in novel *Coxiella* spp. GEM (e.g., bin ID: 3300021338_21) with isolate *C. burnetii icm-dot* CDS. Genes are shown as arrows and colored by predicted function: blue, T4SS components; gray, conserved hypothetical proteins; red, IS111 element transposase, black, response regulator. Majority of the *Coxiella* spp. operon is located on a single scaffold (Scaffold ID: Ga0213839_1000116). Dotted lines connect this scaffold to two disparate genomic scaffolds (Ga0213839_1000281 and Ga0213839_1000243) containing putative orthologs of the remainder of the T4SS components. Two copies of DotA are detected in the GEM. Additional GEMs include 3300021341_23 and 3300021343_16.

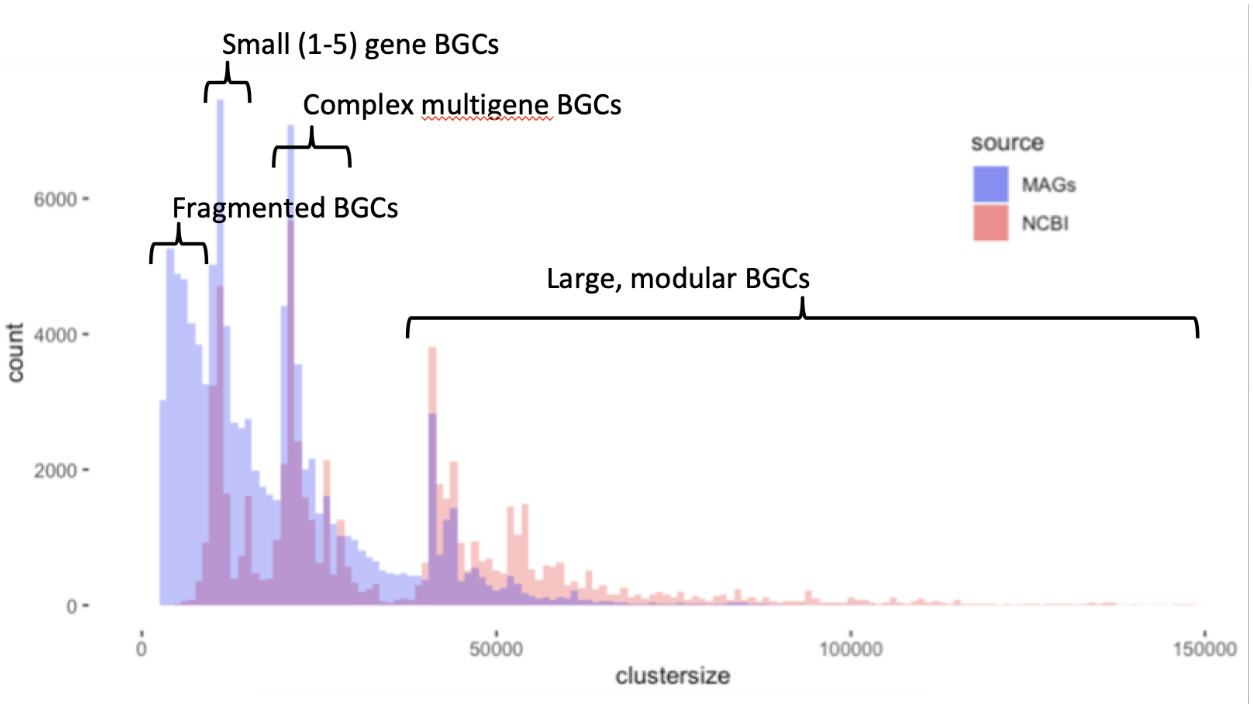

Figure S12. Histogram of BGC sizes from GEM catalogue and NCBI complete genomes. The GEM catalogue contains similar counts for small 1-5 gene BGCs and more complex multi-gene BGCs, but fewer very large (40-100 kb) BGC systems, most of which in that size range are dominated by modular PKS and NRPS genes.

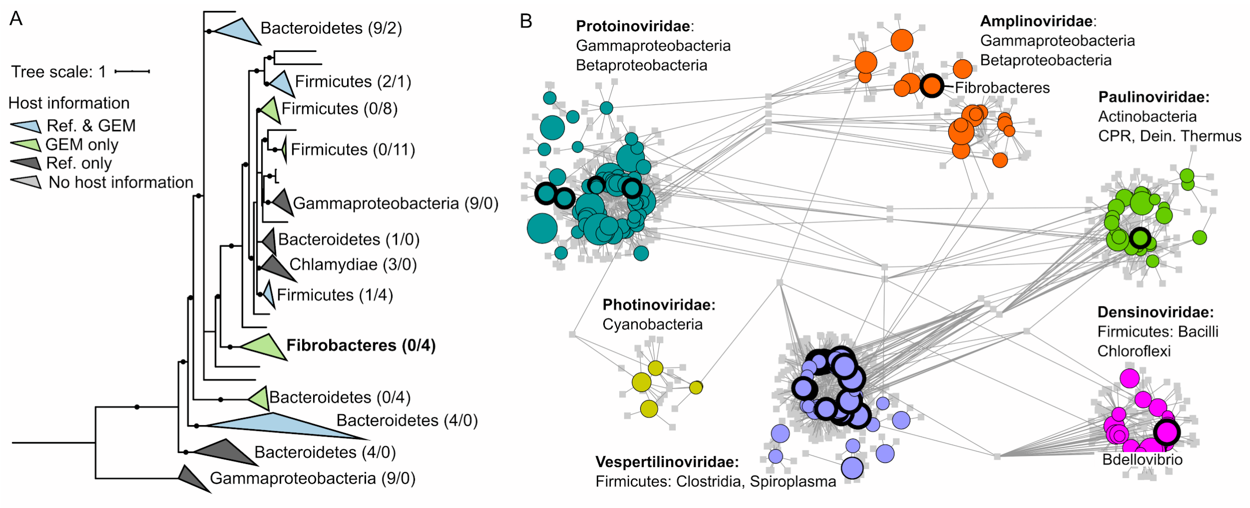

Figure S13. Host information derived from the GEM catalogue for viruses in the *Microviridae* and *Inoviridae* families. A) *Microviridae* phylogeny with associated host information. For each clade of 3 or more virus sequences associated with the same host group, the main host is indicated next to the clade along with the number of sequences linking this *Microviridae* clade to this host group, first in the reference sequences, then in the GEM dataset. Clades are colored according to the origin of the host information, and new host groups identified only from the GEM catalogue are highlighted in bold. All nodes with < 50%

support values are displayed as multifurcation, and nodes with > 80% support are indicated with a black dot. B) Subfamily-level representation of the *Inoviridae* diversity as a genome network. The original network was previously generated in [2]. Briefly, subfamilies are represented by circle nodes and connected to protein clusters, represented as square nodes. Subfamilies are grouped by family, which are indicated on the network along with the major host associated with this family. Subfamilies for which host information could be obtained only from the GEM catalogue are highlighted with a bold outline and linked to the specific GEM-derived host prediction when these host groups had not been associated with inoviruses yet (Fibrobacteres).

References

1. Parks, D.H., et al., *Recovery of nearly 8,000 metagenome-assembled genomes substantially expands the tree of life.* Nat Microbiol, 2017. **2**(11): p. 1533-1542.
2. Khosla, C., et al., *Assembly line polyketide synthases: mechanistic insights and unsolved problems.* Biochemistry, 2014. **53**(18): p. 2875-83.
3. Marahiel, M.A., *A structural model for multimodular NRPS assembly lines.* Nat Prod Rep, 2016. **33**(2): p. 136-40.
4. Udwary, D.W., et al., *Genome sequencing reveals complex secondary metabolome in the marine actinomycete Salinispora tropica.* Proc Natl Acad Sci U S A, 2007. **104**(25): p. 10376-81.
5. Penn, K., et al., *Genomic islands link secondary metabolism to functional adaptation in marine Actinobacteria.* ISME J, 2009. **3**(10): p. 1193-203.
6. Overbeek, R., et al., *The SEED and the Rapid Annotation of microbial genomes using Subsystems Technology (RAST).* Nucleic Acids Res, 2014. **42**(Database issue): p. D206-14.
7. Henry, C.S., et al., *High-throughput generation, optimization and analysis of genome-scale metabolic models.* Nat Biotechnol, 2010. **28**(9): p. 977-82.
8. Arkin, A.P., et al., *KBase: The United States Department of Energy Systems Biology Knowledgebase.* Nat Biotechnol, 2018. **36**(7): p. 566-569.
9. Kanehisa, M. and S. Goto, *KEGG: Kyoto Encyclopedia of Genes and Genomes.* Nucleic Acids Research, 2000. **28**(1): p. 27-30.
10. Latendresse, M., *Efficiently gap-filling reaction networks.* BMC Bioinformatics, 2014. **15**: p. 225.
11. Chen, I.A., et al., *IMG/M v.5.0: an integrated data management and comparative analysis system for microbial genomes and microbiomes.* Nucleic Acids Res, 2019. **47**(D1): p. D666-D677.
12. Parks, D.H., et al., *A proposal for a standardized bacterial taxonomy based on genome phylogeny.* bioRxiv, 2018.
13. Tatusov, R.L., et al., *The COG database: a tool for genome-scale analysis of protein functions and evolution.* Nucleic Acids Res, 2000. **28**(1): p. 33-6.
14. Eddy, S.R., *Accelerated Profile HMM Searches.* PLoS Comput Biol, 2011. **7**(10): p. e1002195.
15. Katoh, K. and D.M. Standley, *MAFFT multiple sequence alignment software version 7: improvements in performance and usability.* Mol Biol Evol, 2013. **30**(4): p. 772-80.

16. Criscuolo, A. and S. Gribaldo, *BMGE (Block Mapping and Gathering with Entropy): a new software for selection of phylogenetic informative regions from multiple sequence alignments.* BMC Evol Biol, 2010. **10**: p. 210.
17. Nguyen, L.T., et al., *IQ-TREE: a fast and effective stochastic algorithm for estimating maximum-likelihood phylogenies.* Mol Biol Evol, 2015. **32**(1): p. 268-74.
18. Hoang, D.T., et al., *UFBoot2: Improving the Ultrafast Bootstrap Approximation.* Mol Biol Evol, 2018. **35**(2): p. 518-522.
19. Letunic, I. and P. Bork, *Interactive Tree Of Life (iTOL) v4: recent updates and new developments.* Nucleic Acids Res, 2019. **47**(W1): p. W256-W259.
20. El-Gebali, S., et al., *The Pfam protein families database in 2019.* Nucleic Acids Res, 2019. **47**(D1): p. D427-D432.
21. Capella-Gutierrez, S., J.M. Silla-Martinez, and T. Gabaldon, *trimAl: a tool for automated alignment trimming in large-scale phylogenetic analyses.* Bioinformatics, 2009. **25**(15): p. 1972-3.
22. Kalyaanamoorthy, S., et al., *ModelFinder: fast model selection for accurate phylogenetic estimates.* Nat Methods, 2017. **14**(6): p. 587-589.
