# Supplementary material for: A genomic catalog of Earth’s microbiomes
Source: Nat Biotechnol. 2020 Nov 9;39(4):499–509. doi: 10.1038/s41587-020-0718-6 (PMC8041624; doi:10.1038/s41587-020-0718-6)
Supplement: Supplementary file 2 — Reporting Summary [file 41587_2020_718_MOESM2_ESM.pdf]

nature research

Corresponding author(s): Stephen Nayfach, Simon Roux, Rekha Seshadri, Daniel Udwary, Neha Varghese, Frederik Schulz, Dongying Wu, David Paez-Espino, I-Min Chen, Marcel Huntemann, Krishna Palaniappan, Joshua Ladau, Supratim Mukherjee, T.B.K. Reddy, Torben Nielsen, Edward Kirton, José P. Faria, Janaka N. Edirisinghe, Christopher S. Henry, Sean P. Jungbluth, Dylan Chivian, Paramvir Dehal, Elisha M. Wood-Charlson, Adam P. Arkin, Susannah Tringe, Axel Visel, IMG/M Data Consortium, Tanja Woyke, Nigel J. Mouncey, Natalia N. Ivanova, Nikos C. Kyrpides, Emiley A. Eloe-Fadrosh

Last updated by author(s): Sep 18, 2020

Reporting Summary

Nature Research wishes to improve the reproducibility of the work that we publish. This form provides structure for consistency and transparency in reporting. For further information on Nature Research policies, see Authors & Referees and the Editorial Policy Checklist.

Statistics

For all statistical analyses, confirm that the following items are present in the figure legend, table legend, main text, or Methods section.

n/a	Confirmed	
☐	☒	The exact sample size (*n*) for each experimental group/condition, given as a discrete number and unit of measurement
☐	☒	A statement on whether measurements were taken from distinct samples or whether the same sample was measured repeatedly
☐	☒	The statistical test(s) used AND whether they are one- or two-sided<br>*Only common tests should be described solely by name; describe more complex techniques in the Methods section.*
☐	☒	A description of all covariates tested
☐	☒	A description of any assumptions or corrections, such as tests of normality and adjustment for multiple comparisons
☐	☒	A full description of the statistical parameters including central tendency (e.g. means) or other basic estimates (e.g. regression coefficient) AND variation (e.g. standard deviation) or associated estimates of uncertainty (e.g. confidence intervals)
☒	☐	For null hypothesis testing, the test statistic (e.g. *F*, *t*, *r*) with confidence intervals, effect sizes, degrees of freedom and *P* value noted<br>*Give P values as exact values whenever suitable.*
☒	☐	For Bayesian analysis, information on the choice of priors and Markov chain Monte Carlo settings
☒	☐	For hierarchical and complex designs, identification of the appropriate level for tests and full reporting of outcomes
☒	☐	Estimates of effect sizes (e.g. Cohen's *d*, Pearson's *r*), indicating how they were calculated

Our web collection on statistics for biologists contains articles on many of the points above.

Software and code

Policy information about availability of computer code

Data collection	We used 10,450 metagenomic assemblies from the IMG/M database (https://img.jgi.doe.gov/) that correspond to 527 studies and 10,331 samples from a myriad of microbial environments (Table S1). Additionally, 564,467 reference genomes were obtained from a variety of sources, including: IMG (59,047 isolates, 8,412 MAGs, 7,066 SAGs), NCBI RefSeq (release 93; 151,730 isolates), GenBank (29,127 MAGs, 1,555 SAGs), and human-associated MAGs from three recent studies (307,530) [4-6].
Data analysis	The following software was used: MetaBAT v0.32.4 and v0.32.5; RefineM v0.0.20; Last aligner v876; CheckM v1.0.11; tRNA-scanSE v2.0; Infernal v1.1.2; Bowtie v2.3.2; MUMMer v.4.0.0; HMMER v3.1b2; FAMSA v.1.2.5; FastTree v2.1.10; scikit-learn v0.21.3; AntiSMASH v5.1; NCBI blast+ v2.9; VirSorter v1.0.3; MAFFT v7.407; trimAl v1.4.rev15; IQ-TREE v1.5.5; iTol v5

For manuscripts utilizing custom algorithms or software that are central to the research but not yet described in published literature, software must be made available to editors/reviewers. We strongly encourage code deposition in a community repository (e.g. GitHub). See the Nature Research guidelines for submitting code & software for further information.

Data

Policy information about availability of data

All manuscripts must include a data availability statement. This statement should provide the following information, where applicable:

- Accession codes, unique identifiers, or web links for publicly available datasets
- A list of figures that have associated raw data
- A description of any restrictions on data availability

All available metagenomic data, bins, and annotations are available through the IMG/M portal (https://img.jgi.doe.gov/). Bulk download for the 52,515 MAGs is available here: https://genome.jgi.doe.gov/GEMs. Genome-scale metabolic models for the non-redundant, high quality GEMs are available in KBase (https://narrative.kbase.us/#org/jgimags). IMG identifiers of all metagenomes binned, including detailed information for each metagenome is available in Supplementary Table S1.

Field-specific reporting

Please select the one below that is the best fit for your research. If you are not sure, read the appropriate sections before making your selection.

☐ Life sciences ☐ Behavioural & social sciences ☒ Ecological, evolutionary & environmental sciences

For a reference copy of the document with all sections, see nature.com/documents/nr-reporting-summary-flat.pdf

Ecological, evolutionary & environmental sciences study design

All studies must disclose on these points even when the disclosure is negative.

Study description	Here we applied genome-resolved metagenomics at scale, to recover 52,515 medium- and high-quality metagenome-assembled genomes (MAGs) from 10,450 metagenomes representing diverse habitats including ocean and other aquatic environments, human- and animal-host associated, and natural and agricultural soils (Figure 1). These 52,515 MAGs form the Genomes from Earth's Microbiomes (GEM) catalogue.
Research sample	We used 10,450 publicly-available metagenomic assemblies from the IMG/M database that correspond to 527 studies and 10,331 samples from a myriad of microbial environments.
Sampling strategy	Metagenome-assembled genomes (MAGs) were screen for quality using standard estimates of completeness and contamination, and dereplicated based on an estimate of average nucleotide identity and described species affiliation. The Minimum Information about Metagenome-Assembled Genomes (MAGs) standards were applied.
Data collection	Not applicable.
Timing and spatial scale	Not applicable.
Data exclusions	Low quality genomes were excluded using the Minimum Information about Metagenome-Assembled Genomes (MAGs) standards criteria. These genomes were excluded as they may negatively impact the quality of the data interpretation and inferred phylogeny.
Reproducibility	All software versions and bioinformatics pipelines are documented for reproducibility.
Randomization	As a data resource, randomization was not applicable.
Blinding	As a data resource, randomization was not applicable.

Did the study involve field work? ☐ Yes ☒ No

Reporting for specific materials, systems and methods

We require information from authors about some types of materials, experimental systems and methods used in many studies. Here, indicate whether each material, system or method listed is relevant to your study. If you are not sure if a list item applies to your research, read the appropriate section before selecting a response.

Materials & experimental systems

n/a	Involved in the study
☒	☐ Antibodies
☒	☐ Eukaryotic cell lines
☒	☐ Palaeontology
☒	☐ Animals and other organisms
☒	☐ Human research participants
☒	☐ Clinical data

Methods

n/a	Involved in the study
☒	☐ ChIP-seq
☒	☐ Flow cytometry
☒	☐ MRI-based neuroimaging
